# Geminiviruses and their satellites: genomic diversity, evolutionary insights, and emerging models

**DOI:** 10.3389/fmicb.2026.1947355

**Published:** 2026-10-12

**Authors:** Jannat Tahir, Muhammad Mubin, Nazia Nahid, Palwasha Kanwal, Saba Shahid Gill, Muhamad Naeem Sattar, Muhammad Amir Qureshi, Eui-Joon Kil, Muhammad Shah Nawaz-ul-Rehman

**Affiliations:** 1 Virology Lab, Centre of Agricultural Biochemistry and Biotechnology, University of Agriculture, Faisalabad, Pakistan; 2 Department of Bioinformatics and Biotechnology, Government College University Faisalabad, Faisalabad, Pakistan; 3 Central Laboratories, King Faisal University, Al-Ahsa, Saudi Arabia; 4 Agricultural Research Institute, Gyeongkuk National University, Andong, Republic of Korea; 5 Department of Plant Medicals, Gyeongkuk National University, Andong, Republic of Korea

**Keywords:** begomoviruses, DNA satellites, geminiviruses, genome plasticity, host–virus interactions, molecular evolution, recombination, RNA-silencing suppression

## Abstract

Geminiviruses infect a wide range of economically important crops, including tomato, cassava, cotton, common bean, and maize, and are characterized by twinned icosahedral particles containing circular single-stranded DNA (ssDNA) genomes. Their genomes have undergone extensive diversification through mutation, recombination and association with satellite molecules. Depending on the genus, geminiviruses are transmitted by hemipteran vectors, including whiteflies, leafhoppers, aphids, and treehoppers, and replicate in plant cell nuclei using host replication machinery, which complicates disease management. Recent taxonomic updates by the International Committee on Taxonomy of Viruses (ICTV) in MSL41 recognize 549 species distributed across 15 genera, with *Begomovirus* accounting for most of the documented species diversity and *Mastrevirus* representing the second-largest genus. Analysis of documented records across eight geographic regions revealed the highest documented geminivirus species richness in the Indian subcontinent. Current evidence indicates that geminivirus diversification is shaped by multiple interacting evolutionary mechanisms, with recombination and modular genetic exchange contributing substantially to the formation of mosaic genomes, while host compatibility and vector ecology impose important ecological and functional constraints on their persistence and diversification. The discovery of previously unrecognized geminivirus lineages, endogenous geminivirus-related sequences, and additional genomic diversity through high-throughput sequencing indicates that the currently recognized diversity of geminiviruses remains incomplete. The evolutionary origin of geminiviruses remains partly unresolved because replication-associated and capsid-associated components show distinct evolutionary histories. Based on comparative genomic, phylogenetic, and recombination evidence, we propose a modular evolutionary model in which replication-associated and capsid-associated components followed distinct evolutionary trajectories, followed by genome-level assembly and diversification associated with the emergence of bipartite genome organization. Overall, the emerging view is that geminiviruses represent genetically and ecologically dynamic lineages shaped by interacting evolutionary processes rather than a single evolutionary trajectory. Broader sampling of hosts and vectors, comparative genomics and functional validation will be essential for resolving their origin and evolutionary relationships.

## 1 Introduction

The family *Geminiviridae* consists of diverse group of plant viruses categorized as small circular single stranded DNA (ssDNA) genomes encapsidated within twinned icosahedra particles (Hipp et al., 2017). Their distinctive virions are composed of two incomplete icosahedral halves, resulting into a geminate structure. This architecture enables packaging of relatively large circular ssDNA genomes within a compact capsid (Hesketh et al., 2018). Geminiviruses infect a wide range of cultivated and non-cultivated monocotyledonous and dicotyledonous plants and are transmitted by different hemipteran vectors. Their biological success is closely associated with their compact ssDNA genomes, dependence on host DNA replication machinery, diverse genome organizations, vector-mediated transmission, and capacity for genetic exchange and association with satellite molecules.

Despite their compact genomes, geminiviruses show substantial variation in c genome organization and coding capacity, ranging from monopartite genomes encoding a limited set of conserved proteins to bipartite genomes with distinct DNA-A and DNA-B components, with genus-specific differences in the number and arrangement of predicted open reading frames (ORFs) (Supplementary Table S1). They exhibit conserved replication mechanism, movement, encapsidation and target the host defense system through overlapping and compact genomic arrangements (Chen et al., 2020; Shakir et al., 2023; Zhang et al., 2023). Whereas, lineage-specific changes in genomic architecture provides opportunities for functional innovation. Together, the compact genomes and plasticity make *Geminiviridae* an important informative system for understanding how the genome organization influences viral diversification.

Although mutation provides the underlying genetic variation, recombination, component capturing or exchange are major contributors to geminivirus diversification, while host and vector adaptation shape the persistence and spread of emerging variants. Recombination can result in chimeric genomes and alter the evolutionary mechanism of individual genomic region. Their functional capacity is expanded due to acquisition of accessory components. While satellites association provides an additional layer of genomic plasticity by influencing pathogenicity and host adaptation. Increasing application of sophisticated techniques like next-generation sequencing and molecular cloning have further revealed previously unrecognized virus diversity including endogenous viral elements and complex evolutionary relationships.

This narrative review was based on published primary research articles, relevant review articles, and authoritative taxonomic resources selected to provide broad coverage of geminivirus taxonomy, genome organization, diversity, satellites, recombination, host–virus interactions, vector associations, and evolutionary origins. Primary studies were prioritized for newly characterized genera, genomic features, satellite associations, recombination events, and evolutionary hypotheses, whereas earlier seminal studies were retained where they provided foundational evidence or established major evolutionary models. Taxonomic assignments and species counts were checked against the International Committee on Taxonomy of Viruses (ICTV) Master Species List 41 (MSL41) and the Virus Metadata Resource (VMR_MSL41.v1.20260729). For the geographic richness analysis, records were retrieved from NCBI Virus and mapped to ICTV taxonomy using the procedure described in Supplementary Data Sheet S1.

Specifically, this review addresses three major questions: (i) what is the current genomic and taxonomic diversity of Geminiviridae and its associated satellites? (ii) What evolutionary and ecological processes contribute to geminivirus diversification, and what geographic patterns characterize their documented diversity? and (iii) what models best explain the evolutionary origins of geminivirus genome components and the emergence of bipartite genome organization?

## 2 Genomic and evolutionary diversity across *Geminiviridae*

Members of the family *Geminiviridae* exhibit remarkable diversity in genome organization, host range, vector specificity, and evolutionary adaptation. Advances in molecular characterization and phylogenetic analysis have substantially refined the taxonomy of this family over the past several decades. Comparative and taxonomic studies of *Geminiviridae* have been further facilitated by development of dedicated genomic resources. GPIBase^1^ is a *Geminiviridae*-plant-insect database that integrates viral sequences information with host and vector associations (Wang et al., 2023). Current classification reflects not only genomic architecture and vector associations, but also the evolutionary diversification and ecological specialization of distinct viral lineages. According to recent ICTV taxonomic revisions, the family currently comprises 15 recognized genera: *Becurtovirus, Begomovirus, Capulavirus, Citlodavirus, Curtovirus, Eragrovirus, Grablovirus, Maldovirus, Mastrevirus, Mulcrilevirus, Opunvirus, Topilevirus, Topocuvirus, Turncurtovirus, and Welwivirus* (ICTV, 2025; ICTV, 2026). These genera are differentiated primarily by host range (monocots or dicots) and their associated insect vectors, including whiteflies, leafhoppers, aphids, and treehoppers. Most genera possess monopartite genomes consisting of a single circular ssDNA molecule of approximately 2.5–3.6 kb. In contrast, some begomoviruses exhibit bipartite genomes in which the genetic material is divided between DNA-A and DNA-B components, which share sequence similarity only within an approximately 200-bp common region (CR) (Rojas et al., 2005; Saccardo et al., 2011; Kraberger et al., 2017; Kumar, 2019; Zhao et al., 2019; Navas-Castillo and Fiallo-Olivé, 2021; Roumagnac et al., 2022; Kumar et al., 2024).

In all genera, viral gene expression is bidirectional and occurs on both the virion-sense (V-sense) and complementary-sense (C-sense) strands. V-sense strand genes generally encode the capsid protein (CP/V1/AV1), a major structural determinant of vector specificity, along with movement-associated proteins such as MP/V2/AV2. In contrast, C-sense strand genes encode the replication-associated protein (Rep/C1/AC1), transcriptional activator protein (TrAP/C2/AC2), replication enhancer protein (REn/C3/AC3), and pathogenicity factor C4/AC4, which regulates host defenses and cell cycle control. In addition, bipartite viruses encode nuclear shuttle protein (NSP/BV1) and movement protein (MP/BC1) on DNA-B (Figure 1). Beyond these canonical ORFs, some members of the family possess additional small ORFs for regulatory and pathogenicity-related functions. For-example, in case of tomato yellow leaf curl virus (TYLCV), C5 protein has been experimentally characterized as pathogenicity determinant and RNA silencing suppressor (Zhao et al., 2022). Although most of these genera employ the conventional 5′-TAATATTAC-3′ pattern at the replication origin, *Becurtovirus* and *Eragrovirus* are significant outliers, utilizing a distinct 5′-TAAGATTCC-3′ sequence. Moreover, the regulatory non-coding genomic segments known as the long intergenic region (LIR) and short intergenic region (SIR) are present in many genera. All geminiviruses replicate through a rolling-circle replication (RCR) mechanism (Rojas et al., 2005; Saccardo et al., 2011; Varsani et al., 2014b; Kumar, 2019; Roumagnac et al., 2022; Lateef et al., 2026).

**Figure 1:**
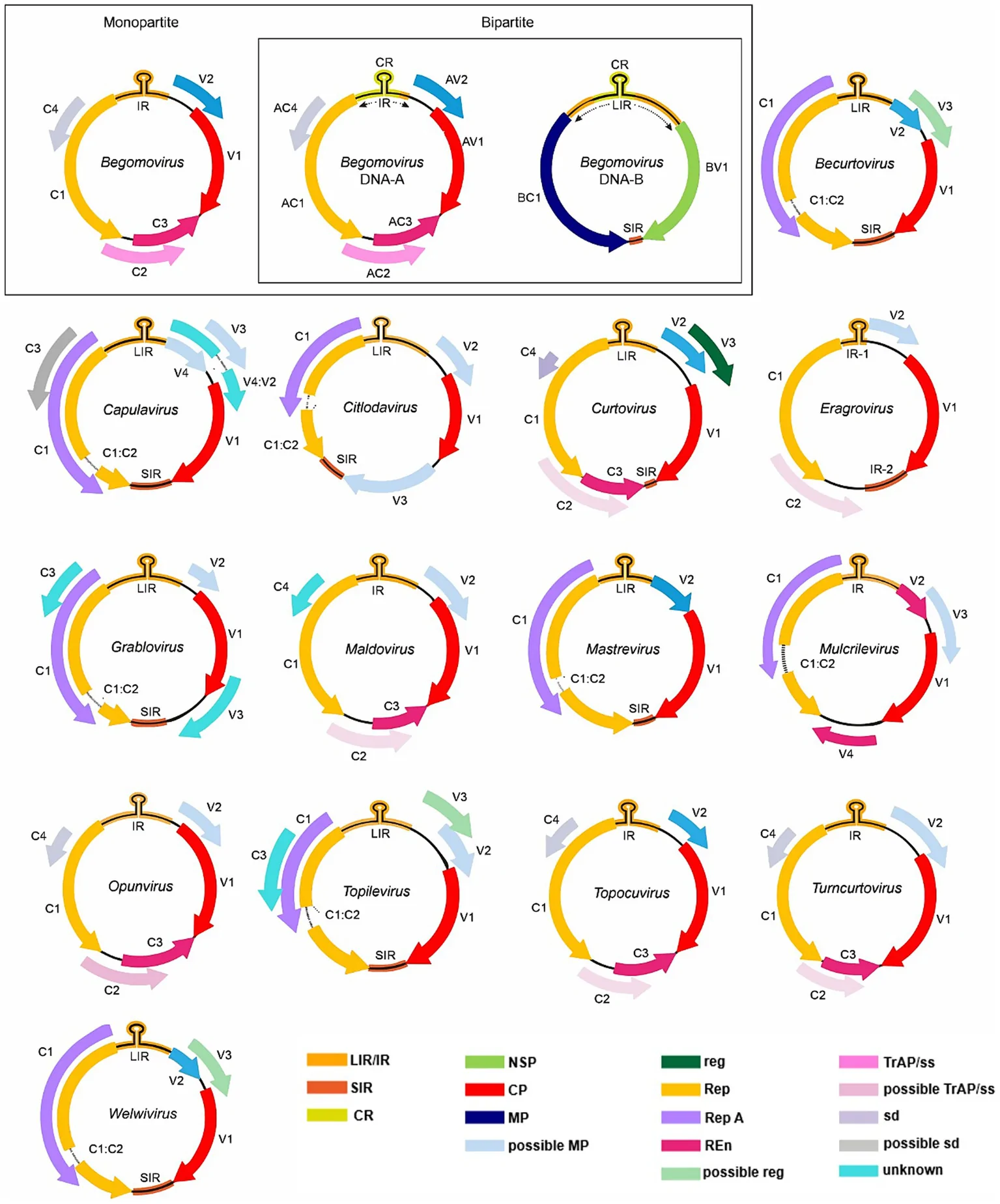
Genomic architecture of 15 genera of the family *Geminiviridae*. The genome maps are adapted and redrawn from ICTV report (https://ictv.global/report/chapter/geminiviridae/geminiviridae). ORFs are classified according to their orientation on virion-sense or complementary-sense strands, and functional categories are shown by different colors. The stem-loop containing the conserved 5’-TAATATTAC-3’ sequence at the origin of replication is shown within the intergenic region (IR/IR-1) or long intergenic region (LIR), where applicable. CR, common region; NSP, nuclear shuttle protein; CP, coat protein; MP, movement protein; reg, regulatory gene; Rep, replication-associated protein; RepA, replication-associated protein A; REn, replication enhancer protein; TrAP, transcriptional activator protein; ss, silencing suppressor; sd, symptom determinant; possible TrAP/ss, ORFs with putative function as either a transcriptional activator protein or a silencing suppressor; possible reg, ORFs with a putative regulatory function; possible sd, ORFs with a putative symptom determinant function. Genome maps are schematic and are not drawn to scale. The *Welwivirus* genome map was drawn based on Debat and Bejerman (2022) and Bejerman and Debat (2023). The ORF annotations for *Mulcrilevirus* were updated based on Han et al. (2024) and Yin et al. (2024).

Among the recognized *Geminiviridae* species by ICTV, *Begomovirus* is largest genus, comprising 466 species and accounting for approximately 84% of species richness of the family. *Mastrevirus* comprises 55 recognized species and it accounts for approximately 10% of total species richness. The proportion of remaining genera each account for approximately 0.2–1% of the recognized species. Thus, the documented species composition of *Geminiviridae* is strongly concentrated in *Begomovirus* and to a lesser extent, *Mastrevirus* (Figure 2; Supplementary Table S1; Briddon et al., 1996; Rojas et al., 2005; Varsani et al., 2009; Loconsole et al., 2012; Liang et al., 2015; Kamali et al., 2016; Al Rwahnih et al., 2017; Varsani et al., 2017; Zerbini et al., 2017; Hasanvand et al., 2018; Fontenele et al., 2020; Fiallo-Olivé et al., 2021; Roumagnac et al., 2022; Flasco et al., 2023; Wang et al., 2023; ICTV, 2025; ICTV, 2026).

**Figure 2:**
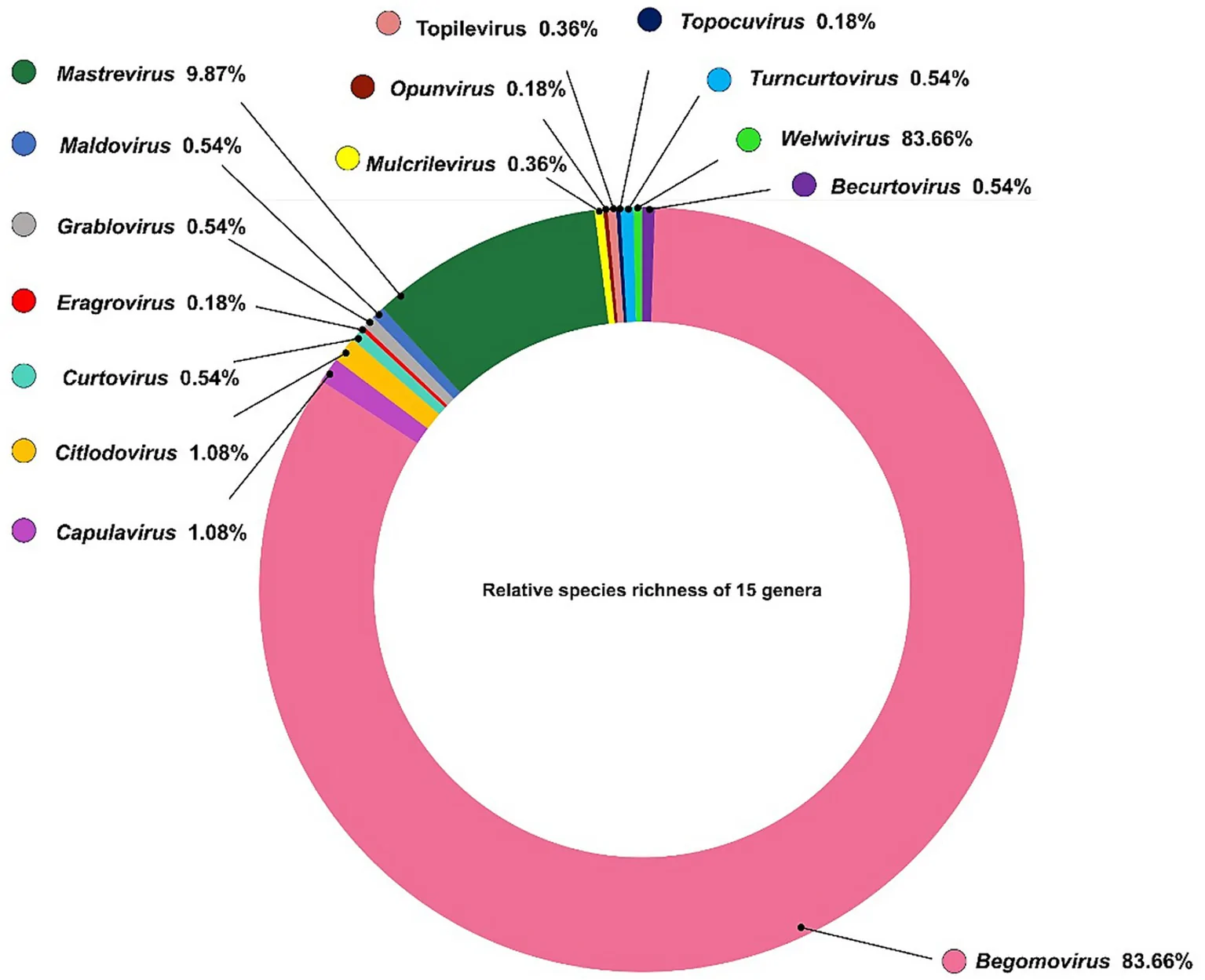
Species composition of *Geminiviridae* by genus. The doughnut chart shows the relative species richness of the 15 recognized genera based on the number of ICTV-recognized species assigned to each genus. Species counts were obtained from ICTV Master Species List 41 (MSL41) and the ICTV Virus Metadata Resource (VMR) (ICTV, 2025; ICTV, 2026). Percentages are based on the total number of recognized species and are rounded for display.

Viruses belonging to different geminiviurses genera exhibit distinct vector association for their transmission. For example, *Becurtovirus*, *Eragrovirus*, *Curtovirus*, *Turncurtovirus* and *Mastrevirus* are predominantly spread by leafhoppers (Rojas et al., 2005; Vanderschuren et al., 2007; Varsani et al., 2014b; Kumar, 2019; Roumagnac et al., 2022; Lateef et al., 2026). It is hypothesized that they use leafhoppers as their vectors (Bennett and Agbandje-McKenna, 2020; Lateef et al., 2026). Mastreviruses primarily infect monocots but also affect certain dicots and are transmitted by leafhoppers (Rojas et al., 2005; Nahid et al., 2008; Kraberger et al., 2013). Maldoviruses infect both host types and frequently remain asymptomatic. There is no known vector for maldoviruses (Roumagnac et al., 2022; Lateef et al., 2026). Consequently, species classifications are based on virion structure, gene complements specific to each strand, sequences of replication origins, and specificity of insect vectors.

From an evolutionary standpoint, the host range across monocots and dicots demonstrates profound lineage specialization influenced by vector ecology, recombination, and host molecular compatibility (Lefeuvre et al., 2009; Lefeuvre et al., 2019; Lateef et al., 2026). Genera infecting monocots, such as *Mastrevirus* and *Eragrovirus*, are predominantly associated with grass-associated hosts and their host associations may reflect lineage-specific ecological adaptations (Varsani et al., 2009; Kumar et al., 2024), whereas mostly dicot-infecting genera developed in conjunction with broadleaf plant radiations. Vector fidelity serves as a strong ecological barrier that maintains genus boundaries as viruses infrequently transition between hosts without concurrent adaptation to a new insect vector. The distinction between whitefly-transmitted begomoviruses and leafhopper- or treehopper-associated genera reflects differences in genome organization, function compactness, and host-vector associations. The diversification of *Begomovirus* reflects the interaction of broad host associations, genome organization, frequent recombination and genetic exchange, and efficient whitefly-mediated transmission, with their relative contributions varying among lineages and ecological contexts. In contrast, *Mastrevirus* and other leafhopper-transmitted genera have retained monopartite genomes, where functional complexity is achieved through nested genes, alternative splicing, multifunctional proteins, or interspecific recombination. Nevertheless, certain other genera like *Grablovirus*, *Citlodavirus*, *Becurtovirus*, and *Eragrovirus* show that extensive genome expansion can evolve outside begomovirus lineage. Thus, it can be determined that vector ecology acts in conjunction with host compatibility and recombination to shape the genome architecture rather than determining it independently (Pan et al., 2020; Fan et al., 2023).

At the molecular level, successful adaptation to a new host lineage requires the coordinated evolution of viral Rep protein for the recognition of host replication factors and of C4 and TrAP proteins for the inhibition of plant specific immune signals (Seal et al., 2006; Lefeuvre et al., 2010; Kumar, 2019; Beam and Ascencio-Ibáñez, 2020). Such evolutionary changes suggest that frequent recombination and component exchange viral strains produce modular genomes that enable occasional host shifts and the creation of novel species (Seal et al., 2006; Lateef et al., 2026). Therefore, monocot and dicot tropism reflects not only taxonomic classification but also long-term co-adaptation among viruses, vectors, and plant hosts (Rojas et al., 2005; Lal et al., 2022; Roumagnac et al., 2022; Fiallo-Olivé and Navas-Castillo, 2023). This conserved genomic framework provides the basis for understanding the diversification of geminiviruses. It is reflected in the unique genome organization, host ranges, vectors associations and evolutionary histories of the 15 currently recognized genera. Vector associations vary substantially across *Geminiviridae*, with confirmed transmission by whiteflies, leafhoppers, aphids, and treehoppers in different genera, whereas vectors remain unknown or putative for several recently characterized genera (Supplementary Table S1).

### 2.1 Becurtovirus

The genus *Becurtovirus,* established in 2014, represents a distinct lineage within the *Geminiviridae* family and is characterized by a ~ 2.84–2.85 kb chimeric genome architecture (Yazdi et al., 2008; Varsani et al., 2014b). It comprises three recognized species as per Master Species List (MSL41) described by ICTV (ICTV, 2025; ICTV, 2026). The virion-sense ORFs (V1/CP, V2, V3) are curtovirus-like, while the complementary-sense Rep (C1/C2) is most closely related to those of mastreviruses. Furthermore, unlike curtoviruses, the becurtovirus genome does not possess C3 and C4 ORFs (Yazdi et al., 2008). In contrast, becurtoviruses possess a unique nonanucleotide sequence (5′-TAAGATTCC-3′) at the virion-strand origin of replication (*ori*), a feature shared only with the genus *Eragrovirus*. This feature distinguishes eragroviruses and becurtoviruses from all other known geminiviruses and nanoviruses (Yazdi et al., 2008; Varsani et al., 2014b). Beet curly top Iran virus (BCTIV) is the species associated with Iranian isolates ranging from strains A-D whereas spinach curly top Arizona virus (SCTAV) was reported in the United States (Varsani et al., 2014b).

### 2.2 Begomovirus

The genus *Begomovirus* is the largest genus within the family *Geminiviridae* comprising 466 recognized species (ICTV, 2025; ICTV, 2026). It comprises whitefly-transmitted viruses that cause billions of dollars in agricultural losses throughout Africa, Asia, and the Americas (Nigam, 2021; Varma and Singh, 2024). Begomovirus genomes can be monopartite or bipartite (~2.5–2.8 kb per component) with a clear phylogenetic division into Old World (OW), New World (NW), legumovirus, and sweepovirus groups. OW begomoviruses often possess a V2 ORF, which is generally absent from NW group (Rojas et al., 2005; Ha et al., 2008). Their association with three classes of circular ssDNA satellites (namely, alpha-, beta-, and deltasatellites) is a key evolutionary feature. These DNA satellites are partially or fully dependent on the helper begomoviruses for replication, movement, encapsidation, and transmission. In particular, betasatellites act as major virulence determinants because their association strongly suppresses host gene-silencing machinery (Cui et al., 2005). High mutation rates, frequent recombination, and genetic exchange contribute to the rapid evolution and diversification of begomoviruses (Belabess et al., 2016; Fiallo-Olivé et al., 2019; Ringeera et al., 2025), making them the largest genus within the *Geminiviridae* (ICTV, 2026). In addition, the global spread of the *Bemisia tabaci* vector complex has facilitated the expansion of begomoviruses into new hosts and geographical regions (Briddon et al., 2008; Xu and Yoshida, 2010).

### 2.3 Capulavirus

The genus currently comprises six recognized species, with reported hosts including alfalfa, bean, Plantago, Euphorbia, Trifolium, and beet; aphid transmission has been demonstrated for several species, whereas vectors remain unknown for others (ICTV, 2025). Members possess monopartite genomes (~2.5–2.9 kb) with a complex arrangement of potential MP ORFs on the virion-sense strand. ORFs are expressed as intron-containing transcripts, suggesting that alternative splicing may have contributed to the evolutionary adaptation of these viruses (Bernardo et al., 2013; Varsani et al., 2017; Chehida et al., 2021). Moreover, the Rep protein retains the RCR motifs but lacks the retinoblastoma-binding domain (LXCXE), a feature common to many geminiviruses (Bernardo et al., 2013). These features suggest that capulaviruses represent an ecologically specialized lineage within the *Geminiviridae*. Although they share the conserved virion-sense origin of replication (ori; TAATATTAC), these viruses exhibit an atypical complement of sense-strand ORFs. Another notable feature is their transmission by the aphid *Aphis craccivora*, in contrast to the common whitefly- or leafhopper-mediated mode of transmission in other genera (Varsani et al., 2017; Zerbini et al., 2017). Thus, vector adaptation is another indication of a separate evolutionary pathway and supports the distinct evolutionary position of capulaviruses.

### 2.4 Citlodavirus

The genus *Citlodavirus* comprises six recognized species, associated with diverse hosts including citrus, Camellia, Morus, Passiflora, and other plant hosts (Supplementary Table S1; ICTV, 2025). Representative isolates include citrus chlorotic dwarf-associated virus (CCDaV), which was first reported in Turkey (Loconsole et al., 2012). Citlodaviruses have monopartite circular ssDNA genomes with 5–6 open reading frames (ORFs) in the virion-sense or complementary-sense orientation. Their genomes are approximately 12–30% larger than those of typical monopartite geminiviruses, mainly because of an expanded MP coding region, which ranges between 891 and 921 nt in length (Fiallo-Olivé et al., 2021). This feature may have evolutionary significance, as such larger MPs are more characteristic of bipartite begomoviruses, where the MP (typically 700–1,100 nt) is encoded on the DNA-B component. Other members of this genus include passion fruit chlorotic mottle virus (PCMoV), reported in Brazil (Fontenele et al., 2018) and camellia chlorotic dwarf-associated virus (CaCDaV), reported in China (Zhang et al., 2018).

### 2.5 Curtovirus

Curtoviruses form a phylogenetically differentiated genus within the family *Geminiviridae*, characterized by monopartite circular ssDNA genomes ranging from 2.9 to 3.0 kb and encoding 6–7 genes. These viruses, including beet curly top virus (BCTV), are transmitted by the leafhopper *Circulifer tenellus* and infect more than 300 dicotyledonous hosts, typically causing curly top symptoms. Both intra- and interspecific recombination events have been detected among BCTV, spinach severe curly top virus (SpSCTV), and horseradish curly top virus (HCTV). Recombination breakpoints are mostly clustered at the 3’ ends of V1 and C1, contributing to genetic diversification and strain emergence (Varsani et al., 2014a). This genus is represented by three species (ICTV, 2025).

### 2.6 Grablovirus

Grabloviruses possess unusually large monopartite circular ssDNA genomes (~3.2 kb) with a genomic organization divergent from those of other geminiviruses (ICTV, 2025). Their 6–7 bidirectionally arranged ORFs lack clear homology with other genera, supporting their position as an evolutionarily divergent lineage within the family (Fiallo-Olivé et al., 2021; Flasco et al., 2021; Flasco et al., 2023). The genus comprises three recognized species: grapevine red blotch virus, prunus latent virus, and wild vitis latent virus, infecting woody perennials, including grapevine, *Prunus* spp., and wild *Vitis*, respectively. Laboratory transmission by grafting as well as by the treehopper *Spissistilus festinus* provides insights into its ecological and evolutionary constraints (Flasco et al., 2023).

### 2.7 Mulcrilevirus

The genus *Mulcrilevirus* is highly divergent from other recognized geminiviruses as its members share very low nucleotide sequence identity (<61%) with known geminiviruses (Roumagnac et al., 2022). The genome is monopartite circular ssDNA and contains the standard TAATATTAC origin-of-replication motif. A notable evolutionary feature of mulcrileviruses is Rep expression from an alternatively spliced complementary-sense transcript, a mechanism elucidated through total small RNA (sRNA) and RNA sequencing (RNA-seq) data (Qiu et al., 2020). This genus currently includes two species *Mulcrilevirus broussonetiae* and *Mulcrilevirus mori* (Lu et al., 2015; Ma et al., 2015; Lu et al., 2022; ICTV, 2025). Currently, mulcrileviruses are associated with specific host plants, including *Morus alba* and *Broussonetia papyrifera* in China and are transmitted by the leafhopper vector *Tautoneura mori* (Roumagnac et al., 2022).

### 2.8 Opunvirus

The genus *Opunvirus* is highly divergent from other currently characterized geminiviruses. Its sole recognized member, opuntia virus 1 (OpV1) (ICTV, 2025; ICTV, 2026), was identified via high-throughput sequencing (HTS) from NW. Cactaceae and associated insects. OpV1 possesses a monopartite circular ssDNA genome (~2.9 kb) with the fundamental genomic features of geminiviruses. However, its CP exhibits significant divergence (<65% identity to other genera), and its proposed species demarcation threshold of 78% is notably lower than those used for many other geminivirus genera, supporting its classification as a separate, divergent lineage within the family *Geminiviridae* (Zerbini et al., 2017; Fontenele et al., 2020).

### 2.9 Topilevirus

Among recently established genera, the genus *Topilevirus* (recognized in 2021) provides a notable evolutionary link. It comprises two species, tomato apical leaf curl virus and tomato geminivirus 1 (ICTV, 2025; ICTV, 2026), both identified from tomato in South America (Fontenele et al., 2017; Vaghi Medina et al., 2018). Their monopartite genomes (~2.7 to 3.0 kb) show genomic architecture and bidirectional ORF arrangements similar to those of mastreviruses, capulaviruses, grabloviruses, and citlodaviruses. The genome contains LIR and SIR, respectively. These structural similarities and evidence of spliced Rep transcripts suggest that they share a common evolutionary lineage with these OW geminivirus genera. The absence of any known vector and limited known host range currently restricted to tomato and *Cleome* sp. highlights its distinct ecological position within geminiviruses (Zerbini et al., 2017; Roumagnac et al., 2022).

### 2.10 Topocuvirus

The genus *Topocuvirus* is represented primarily by tomato pseudo-curly top virus (TPCTV) (ICTV, 2025; ICTV, 2026). TPCTV possesses a 2.8-kb circular ssDNA genome, infects dicotyledonous hosts, and is transmitted by treehoppers, notably *Micrutalis malleifera*. The genomic organization of this genus resembles that of curtoviruses and monopartite begomoviruses. Its genome generally encodes six open reading frames (ORFs) and employs the conserved nonanucleotide sequence (5′-TAATATTAC-3′) at the origin of virion-strand replication (Briddon et al., 1996). Topocuviruses are biologically significant because they can trans-complement the systemic movement of the DNA-A components of bipartite begomoviruses. In *Nicotiana benthamiana,* TPCTV efficiently facilitated systemic movement of the DNA-A component of tomato golden mosaic virus (TGMV) and African cassava mosaic virus (ACMV) without their corresponding DNA-B components. This observation highlights substantial molecular compatibility within the plant phloem environment. Notably, trans-complementation was not observed in other examined hosts (Briddon and Markham, 2001).

### 2.11 Turncurtovirus

The genus *Turncurtovirus* was established to accommodate a distinct monopartite geminivirus lineage characterized by a unique genomic structure (Varsani et al., 2014b). Its members possess circular ssDNA genomes of approximately 3.0 kb that encode six ORFs. The genomic organization features a curtovirus-like complementary-sense arrangement (four ORFs) but only two virion-sense ORFs, contrasting with curtoviruses (three) and mastreviruses, which contain two complementary-sense ORFs (Varsani et al., 2014b; Hasanvand et al., 2018). Despite their positional homology with curtoviruses, most of their encoded proteins are highly divergent from curtoviruses. Notably, the V2 protein shows no significant identity to any sequences in the current databases. Further distinguishing features include the presence of a unique iteron-related domain in their Rep protein and the conserved mastrevirus-like nonanucleotide origin sequence. Based on the current ICTV species demarcation threshold (<80% genome-wide pairwise identity), this genus includes three distinct species: *Turncurtovirus brassicae*, *T. rapae* and *T. sesame* (Briddon et al., 2010a; Varsani et al., 2014b; Kamali et al., 2016; Hasanvand et al., 2018; ICTV, 2025; ICTV, 2026).

### 2.12 Eragrovirus

The genus *Eragrovirus* comprises a single species *Eragrostis curvula streak virus* (ECSV) (Varsani et al., 2009; Varsani et al., 2014b; ICTV, 2025; ICTV, 2026). It possesses a chimeric genome with components derived from different geminivirus genera, which is pointing toward its modular evolution. Its coat protein (CP or V1) is most similar to Eurasian mastreviruses whereas its Rep protein is phylogenetically related to those of begomoviruses, curtoviruses, and topocuviruses (Varsani et al., 2009).

### 2.13 Maldovirus

Members of the genus *Maldovirus* are a unique group of monopartite circular ssDNA viruses. Significantly, these viruses have been reported exclusively from the asymptomatic dicotyledonous and monocotyledonous host plants (Liang et al., 2015; Al Rwahnih et al., 2017). The genus currently comprises three recognized species: *apple geminivirus 1, grapevine geminivirus A*, and *juncus maritimus geminivirus 1* (ICTV, 2025; ICTV, 2026). Apple geminivirus (AGV) is a representative member of the genus *Maldovirus,* which derives its name from *Malus domestica* virus because AGV was first reported from apple (*Malus domestica*) in China (Liang et al., 2015). Their genomic organization is like typical monopartite begomoviruses except for the presence of conserved rolling-circle motifs (FLTYP, HLH, YIEKG, GRS) in the Rep protein. The Rep proteins exhibit 28–69% identity with proteins from other geminiviruses, whereas CPs showed 14–29% nucleotide identity. So far, the insect vector for maldoviruses is unknown (Roumagnac et al., 2022; Lateef et al., 2026). Notably, GGVA has been detected alongside ~1.5 kb long components in grapevine collections from the United States, which may represent either subgenomic components or defective forms of the virus. Importantly, these subgenomic or defective components were transmissible together with the full-length GGVA genome (Liang et al., 2015; Al Rwahnih et al., 2017).

### 2.14 Mastrevirus

The genus *Mastrevirus* is the second largest genus in this family as it constitutes 10% of ICTV’s recognized species (ICTV, 2025; ICTV, 2026). Members possess monopartite circular ssDNA genomes (2.6–2.8 kb) with persistent circulative transmission by leafhoppers. Predominantly identified in the OW, they have a narrow host range restricted to monocots (mostly cereals and grass families). However, certain dicot-infecting species such as chickpea chlorotic dwarf virus also occur in the OW. A key genomic feature is the use of transcript splicing, which generates multiple proteins from overlapping ORFs including the complementary-sense proteins Rep and RepA (Rojas et al., 2005; Chen et al., 2015; Varsani et al., 2017). These compact genomic features, coupled with vector-transmission specificity, underscore the role of replication compatibility and insect-vector coevolution in their speciation.

### 2.15 Welwivirus

Among the 15 recognized genera, *Welwivirus* is the most recently established genus of the family *Geminiviridae*. However, this genus remains insufficiently characterized due to limited biological and molecular data. Currently, it includes only two recognized species, *welwivirus welwitschiae* and *W. mirabilis* (ICTV, 2025). Phylogenetic analyses have shown that their Rep proteins cluster with those of *Capulavirus* and *Topilevirus,* whereas their CP proteins cluster with those of *Mastrevirus*. This phylogenetic incongruence is consistent with possible genetic exchange among lineages related tomastreviuses and capulaviruses.

Despite the considerable diversity among geminivirus genera, several common evolutionary patterns can be observed across the family. Recombination plays a major role in shaping genome diversity, while vector specificity strongly influences host adaptation and transmission dynamics. In addition, the modular nature of geminivirus genomes suggests that distinct functional components may have evolved and assembled progressively over time. The differences between monopartite and bipartite genomes likely reflect adaptive strategies that optimize transmission and host interactions. Collectively, these shared features highlight the highly dynamic and interconnected nature of geminivirus evolution within the *Geminiviridae* family. To facilitate this comparison, salient characteristics of the 15 recognized geminivirus genera are summarized in Supplementary Table S1.

In addition to these recognized genera, candidate, unclassified and geminivirus-like sequences are continuously being identified through bioinformatic reanalysis and targeted molecular screenings. For instance, endogenous geminivirus-like elements (EGV1 and EGV2) have been identified in the nuclear genomes of *Dioscorea* species, and are hypothesized to have integrated approximately 1.4 million years ago. Their Rep-coding sequences provide evidence of ancient begomovirus-like ancestors (Filloux et al., 2015). Such findings indicate that the known breadth of *Geminivirdae* diversity remains undersampled. Moreover, phylogenetic reanalysis of previously recognized sequences has revealed possible taxonomic misclassifications within the family. Recently, “begomovirus spathoglottis 1” and “begomovirus spathoglottis 2” have been proposed for reclassification in the genus *Mastrevirus* (Lateef et al., 2026). These candidate and reclassified sequences are also relevant to understanding geminivirus evolution because they may contain genomic architectures or gene combinations that are poorly represented among currently recognized taxa. Phylogenetic analysis of such sequences can help identify previously unrecognized evolutionary relationships, ancestral genomic components, and possible cases of recombination or modular gene exchange. Thus, their continued characterization may help discriminate between competing models of geminivirus evolution and refine the proposed evolutionary relationships within the family.

## 3 Current understanding of geminivirus-associated satellites

The complex biological and pathogenic properties of many geminiviruses cannot be fully understood without considering their associated DNA satellite molecules. Although these small circular ssDNA satellites are not classified within the family *Geminiviridae*, ICTV classifies them into two separate families, *Alphasatellitidae* and *Tolecusatellitidae*.

Since the first discovery of a subviral component (now known as deltasatellite in the genus *deltasatellite*) with tomato leaf curl virus in Australia in 1997 (Dry et al., 1997), the DNA satellites have been organized into three established classes, alphasatellites, betasatellites, and deltasatellites. Each of these DNA satellites possess unique genomic and evolutionary features (Collmer and Howell, 1992; Simon et al., 2004).

### 3.1 Betasatellites as the pathogenicity determinants

Betasatellites are formally classified within the genus *Betasatellite* in the family *Tolecusatellitidae*, which currently comprises 120 recognized species according to ICTV MSL41. These ~1.3-kb satellite components are primarily found in association with OW monopartite begomovirus complexes (Dry et al., 1997; Briddon and Stanley, 2006; Fiallo-Olivé et al., 2016). Hallmark features of betasatellite genomes include an adenine-rich (A-rich) region on the complementary-sense strand, a conserved stem-loop (hairpin) structure with nonanucleotide motif (5’-TAATATTAC-3’), a satellite conserved region (SCR), and a single gene encoding the βC1 protein (Saunders et al., 2002a; Briddon et al., 2003; Zhou et al., 2003; Saunders et al., 2008).

Betasatellites depend completely on helper virus for their replication and movement (Jose and Usha, 2003; Saunders et al., 2004; Gnanasekaran et al., 2021; Fondong, 2025). The βC1 protein acts as a symptom determinant and pathogenicity factor in helper-virus complexes (Saunders et al., 2000; Saeed et al., 2005; Briddon and Stanley, 2006; Saunders et al., 2008; Patil and Fauquet, 2010). In addition, it acts as a versatile silencing suppressor of host RNA silencing and is involved in disrupting multiple cellular processes (Cui et al., 2005; Saeed et al., 2007). Some studies suggested that betasatellites can functionally substitute for the DNA-B component of bipartite begomoviruses under experimental conditions (Saeed et al., 2007; Patil and Fauquet, 2010). Saeed et al. (2007) showed that a cotton leaf curl associated betasatellite complemented the systemic movement function of DNA-B when co-inoculated with tomato leaf curl New Delhi virus (ToLCNDV) in tomato plants. They showed that inoculation with DNA-A alone and co-inoculation with disrupted βC1 resulted in local infection only. However, this heterologous βC1 virus pairing cannot be generalized to imply that all betasatellites can completely replace DNA-B encoded functions. The mechanistic basis for this complementation also remains poorly understood. One hypothesis involves the DNA-binding specificity of βC1 (Cui et al., 2005). Subsequent studies further support this concept by showing that βC1 interacts with both dsDNA and ssDNA (Gnanasekaran et al., 2019b; Gnanasekaran et al., 2021). Moreover, it has been revealed that ATP influences this binding, with βC1 exhibits reduced DNA-binding ability in the presence of ATP, suggesting a potential interaction between the ATP-binding and DNA-binding functions of the protein (Gnanasekaran et al., 2021). However, no experimental evidence has been found in direct linkage of this DNA binding ability of βC1 to its movement complementation phenotype. Thus, the exact role of βC1 in viral movement and its potential interaction with the helper virus CP still needs to be investigated.

Notably, βC1 is not restricted to the nucleus and also accumulates in the nucleolus (sequence-dependent manner) and chloroplasts, where it may interfere with organellar function and host defense signaling (Cui et al., 2005; Yang et al., 2011a; Bhattacharyya et al., 2015; Gnanasekaran et al., 2019a; Gnanasekaran et al., 2019b). Recently, another ORF, namely βV1 was also reported in case of radish leaf curl betasatellite (RaLCB) (Gupta et al., 2022). This finding expands the functional complexity of betasatellites beyond the traditionally recognized single βC1 gene. This ORF is located on the virion strand and was shown to encode a functional protein involved in pathogenicity and host interactions. Comparative genomic analyses and experimental validation across diverse betasatellites are required to determine whether βV1 is a unique feature of RaLCB or a previously overlooked conserved element in other betasatellite genomes.

### 3.2 Alphasatellites as rep-encoding autonomously replicating satellite components

Alphasatellites have been officially classified within the family *Alphasatellitidae*. Based on their association with helper viruses, geminivirus-associated alphasatellites are categorized under the subfamily, “*Geminialphasatellitinae*.” Based on the current ICTV MSL41 classification (Supplementary Table S1), they are divided into 8 genera and 60 species (Zerbini et al., 2017; Fiallo-Olivé et al., 2021; Lateef et al., 2026). Geminialphasatellites are approximately 1.38 kb in length and encode their own Rep protein. This evolutionary perspective of this feature is the nanovirus-like Rep component, which is presumed to be captured during evolution of these alphasatellites. This feature enables their autonomous replication, but they remain dependent on helper begomoviruses for movement, encapsidation, and transmission. Their biological role is complex and context-dependent: some attenuate disease by reducing betasatellite accumulation (Idris et al., 2014; Kumar et al., 2021), others enhance symptoms, and many encode Rep proteins that suppress host gene silencing (Nawaz-ul-Rehman et al., 2010; Abbas et al., 2019). They were initially considered to occur exclusively in the OW as part of monopartite begomovirus-betasatellite complexes, but recent studies have identified alphasatellites in the NW in association with bipartite begomoviruses (Paprotka et al., 2010; Romay et al., 2010) and, notably, with a wheat-infecting mastrevirus (Kumar et al., 2014). This highlights alphasatellites as a dynamic and versatile group of DNA satellites, with broader evolutionary potential and host adaptability, contradictory to their previous perception as simple accessory molecules with no direct role in disease biology.

Experiments with cassava-infecting begomoviruses have revealed significant variability in their ability to trans-replicate betasatellites and interact with alphasatellites (Patil and Fauquet, 2010). These differential interactions correlate with the modulation of symptom expressions, indicating that satellite acquisition may play a role in the evolution and pathogenic potential of begomovirus complexes.

### 3.3 Deltasatellites as defective derivatives

Deltasatellites represent a distinct class of DNA satellites classified in the genus *Deltasatellite*, family *Tolecusatellitidae*, which currently comprises 13 recognized species according to ICTV MSL41. These ~700-nt molecules are hypothesized to be structural derivatives of betasatellites, likely through loss of the βC1 gene followed by divergence of replication-associated sequences that enabled adaptation to interactions with specific helper begomoviruses (Saunders et al., 2008; Ng et al., 2011; Fiallo-Olivé et al., 2012; Akhtar et al., 2014; Lozano et al., 2016). Although they retain an A-rich region and an SCR-like sequence, they do not encode any ORFs. They exhibit significant divergence, yet their evolutionary origin remains unclear (Ng et al., 2009; Fiallo-Olivé et al., 2012; Lozano et al., 2016). Deltasatellites are fully dependent on their helper begomoviruses for replication, movement, encapsidation, and transmission (Lozano et al., 2016). Their effects are often modulatory; in some cases, they reduce helper viral accumulation and symptom severity, which suggests a regulatory function in virus–host interactions (Fiallo-Olivé et al., 2012; Lozano et al., 2016). This interaction may prolong host viability and enhance transmission opportunities. Initially identified in the OW, deltasatellites have also been found in the NW (Cuba, Spain and Venezuela), in association with sweepoviruses and other begomoviruses (Fiallo-Olivé et al., 2012; Fiallo-Olivé et al., 2016; Lozano et al., 2016).

Satellite association is not restricted to *Begomovirus*. Wheat dwarf India virus (WDIV) was reported from wheat together with three satellites: Cotton leaf curl Multan alphasatellite (CLCuMA), guar leaf curl alphasatellite (GLCuA) and ageratum yellow leaf curl betasatellite (AYLCB), in the absence of any detectable begomovirus molecule (Kumar et al., 2014). Similarly, chickpea chlorotic dwarf virus (CpCDV) was detected in association with CLCuMA and cotton leaf curl Multan betasatellites (CLCuMB) in spinach (Hamza et al., 2018). These observations suggest that satellite-helper virus associations may extend beyond *Begomovirus* and occur across distinct geminivirus lineages. However, the available evidence remains limited to a small number of characterized cases, and the prevalence and evolutionary significance of such associations across *Geminiviridae* remain unresolved.

Alongside the established alpha-, beta- and deltasatellites, a distinct class of satellite-like sequences, termed sequences enhancing geminivirus symptoms (SEGS), has been reported in association with cassava mosaic begomoviruses. Two SEGS sequences, SEGS-1 and SEGS-2, were initially identified in cassava (Maredza et al., 2016). These sequences have been experimentally shown to enhance disease symptoms and encode a small protein of 75-amino acids (Maredza et al., 2016; Rajabu et al., 2023). Unlike alpha-, beta- and deltasatellites, SEGS are reported to be host-derived (Aimone et al., 2021). Moreover, SEGS-2 contains a 26-bp motif with similarity to alphasatellite sequences, suggesting possible evolutionary interaction with the cassava genome (Ndunguru et al., 2016). Unlike alphasatellites, SEGGS lack a Rep protein and depend on the helper virus for replication (Aimone et al., 2021). Their natural occurrence is best documented in cassava associated with cassava mosaic begomoviruses, and evidence for a broader host range remains limited (Maredza et al., 2016; Rajabu et al., 2023). Thus, SEGS should be considered a distinct and comparatively understudied group of geminivirus-associated satellite-like sequences rather than a formally classified satellite genus.

## 4 Evolutionary perspectives and genome plasticity

The origin of multipartite genomes in geminiviruses remains unresolved. Genome fragmentation followed by functional complementation has been proposed as a general mechanism for the evolution of multipartite viruses, but direct evidence for this process in geminiviruses is lacking. For bipartite begomoviruses, specific models have instead proposed either the capture of a satellite-like DNA molecule or the acquisition and subsequent evolution of distinct movement-associated genes (Nawaz-ul-Rehman and Fauquet, 2009; Briddon et al., 2010b). Geminivirus genomic plasticity is reflected in the ability of these viruses to undergo recombination and component exchange (Hou and Gilbertson, 1996; Padidam et al., 1999). These processes can generate alternative genome organizations and combinations of viral and satellite components, although their evolutionary consequences vary among lineages. Geminivirus-associated satellites, particularly betasatellites, can influence disease development, viral accumulation, and host defense responses (Gnanasekaran et al., 2019a). Although these effects may provide selective advantages under particular ecological conditions, direct evidence that satellite acquisition causes host-range expansion remains limited.

There are several non-mutually exclusive hypotheses regarding the origin of DNA-B. It was first proposed by Kikuno et al. (1984) that DNA-A component duplicated to evolve into NSP, followed by the addition of *MP* gene from an unknown source. Evidence presented in favor of this hypothesis was based on reported amino acid sequence similarity between CP and NSP in ACMV (Kikuno et al., 1984). However, the reported CP-NSP sequence similarity was not supported by subsequent sequence analyses using modern software applied to ACMV and a substantially larger dataset of bipartite begomoviruses (Briddon et al., 2010b). Another proposal is that the DNA-B component of bipartite begomoviruses may itself have originated from a captured DNA satellite molecule, illustrating how the assimilation of subviral elements can drive the emergence of new genomic architectures (Nawaz-ul-Rehman and Fauquet, 2009; Briddon et al., 2010b). This hypothesis also postulated the duplication of CP gene to form NSP gene which is consistent with the earlier hypothesis (Kikuno et al., 1984). This satellite capture theory is supported by several circumstantial evidences. Distinct evolutionary histories of DNA-A and DNA-B molecules (Briddon et al., 2010b), natural occurrence of “regulon grafting” mechanism to integrate independent ssDNA molecules (Saunders et al., 2002b), and the ability of betasatellites to provide selective advantage to their helper viruses by overcoming host defense proteins (Cui et al., 2005) or by providing movement function (Saeed et al., 2007). However, the proposed derivation of NSP from the CP of modern begomoviruses has recently been challenged by Cárdenas-Conejo et al. (2025). They hypothesized that NSP evolved from CP encoded by an ancient, unknown non-begomovirus lineage. Newly discovered rhododendron-derived endogenous geminivirus-like elements (rEGVs) may represent remnants of this lineage. Cárdenas-Conejo et al. (2025) supported this scenario using complementary sequence, phylogenetic, and structural evidence. Homology searches identified significant similarities between NSP proteins encoded by rhododendron-derived endogenous geminivirus-like DNA-B elements and CP proteins encoded by corresponding DNA-A elements and selected geminivirus lineages. Comparative conservation analyses further support shared sequence features, while structural analyses indicated that NSP and CP adopt a related jelly-roll architecture. Together with the phylogenetic placement of these rhododendron-derived elements, these observations support the hypothesis of an ancient CP origin for NSP distinct from modern begomovirus CPs. However, because the proposed ancestral transition cannot be directly observed, the evidence should be considered strong comparative support for an evolutionary model rather than direct experimental proof (Cárdenas-Conejo et al., 2025). In a complementary context, Butkovic et al. (2023) proposed a hypothesis for the evolution of 30 K MPs, which includes geminivirus MP/BC1, from the jelly-roll domain of CP of plant-infecting small RNA and DNA viruses. They proposed that duplication of this CP gene in an ancestral plant virus was followed by neofunctionalization of one CP copy into 30 K MPs. Structural and sequence-based comparisons identified conserved similarities between 30 K MPs and single-jelly roll CPs, with particularly close relationships to CPs of *Bromoviridae* and *Geminiviridae*. These findings supported the hypothesis that 30 K MPs originated through duplication or horizontal acquisition of an ancestral CP followed by neofunctionalization (Butkovic et al., 2023). Thus, the findings of Cárdenas-Conejo et al. (2025) and Butkovic et al. (2023) should not be considered competing hypotheses for the origin of DNA-B as a complete genomic component. Rather, they suggest that the two DNA-B-encoded MPs may have distinct evolutionary histories. Under this model, NSP/BV1 could have been derived from a CP of an ancient geminivirus lineage, whereas MP/BC1 could have originated independently through the evolution and horizontal transfer of a 30 K-type MP from an ancestral single-jelly-roll CP. Such a modular origin is compatible with the mosaic evolutionary history observed in viral genomes and suggests that DNA-B may have been assembled through sequential acquisition and functional integration of genes with different evolutionary origins.

The continued discovery and classification of the DNA satellites into new subviral families (*Tolecusatellitidae* and *Alphasatellitidae*) show their fundamental role in the ecology, evolution, and pathogenicity of geminiviruses. Betasatellites show significant replication promiscuity because they can be trans-replicated by different helper viruses even though they lack typical iteron motifs for Rep binding. Deletion mutagenesis studies on ageratum yellow vein virus (AYVV) have showed that *Ori* in betasatellites is ~ 360 nt and confined to the SCR and adjacent upstream sequences (Saunders et al., 2008). Furthermore, recombinant betasatellites found in *Digera arvensis* indicate that the SCR acts as a recombination hotspot that drives mosaic satellite genome evolution (Mubin et al., 2009).

Some of the most compelling evidence for genome plasticity of geminiviruses is found in the formation of experimentally detectable pseudo-recombinants. In India, a supervirulent pseudo-recombinant was produced when the DNA-A component of ToLCNDV was co-inoculated with the DNA-B component of tomato leaf curl Palampur virus (ToLCPalV) in tomato (Kanakala et al., 2013). Likewise, in Brazil, pseudo-recombinants were formed in tomatoes by co-inoculating DNA-A and DNA-B components of tomato rugose mosaic virus (ToRMV) and tomato severe rugose virus (ToSRV). The two viruses were reported to share compatible DNA-B components (Silva et al., 2014).

## 5 Updated perspectives on conserved and variable features of geminiviruses

Building on foundational observations from recent decades, subsequent studies of geminiviruses and DNA satellites have yielded high-resolution structural and genomic insights. These studies have transformed our understanding of geminiviruses by revealing conserved strategies and evolutionary processes that generate genetic diversity across the family *Geminiviridae*. Although RCR is a conserved and well-established feature of *Geminiviridae,* its persistence across highly divergent genera and associated satellite molecules provides an important evolutionary framework for understanding how a conserved replication strategy has been maintained despite extensive diversification in genome organization, Rep proteins, and accessory functions. Structural studies have uncovered a conserved double-ring hexameric architecture of the Rep protein and its conserved catalytic sites responsible for nicking, ligation, and helicase activity (Laufs et al., 1995; Pant et al., 2001; Choudhury et al., 2006; Clérot and Bernardi, 2006; Ruhel and Chakraborty, 2019). Rep proteins have been classified within the broader category of circular Rep-encoding single-stranded DNA (CRESS-DNA) viral proteins (Tarasova and Khayat, 2023). The classification of geminiviruses within the broader CRESS-DNA group highlights their shared Rep-mediated replication mechanism with diverse circular ssDNA viruses (Tarasova and Khayat, 2023). However, this does not mean that geminiviruses are closely related to all CRESS-DNA viruses. Comparative studies show that CRESS-DNA viruses share conserved Rep domains but have diverged substantially across different evolutionary lineages (Zhao et al., 2019; Krupovic et al., 2020). Thus, the CRESS-DNA classification provides a broader evolutionary context for geminivirus Rep proteins but does not fully resolve their evolutionary origin. These proteins are characterized by a conserved HUH endonuclease domain essential for RCR initiation (Tompkins et al., 2021; Tarasova and Khayat, 2023). In addition to catalysis, Rep interacts with host replication factors, including DNA polymerases α and δ, PCNA, and replication protein A, to convert single-stranded viral DNA into double-stranded minichromosomes (Wu et al., 2021; Chi et al., 2025). This strategic recruitment of host cellular machinery represents a deeply conserved replication strategy across all geminivirus genera (Beam and Ascencio-Ibáñez, 2020; Shakir et al., 2023).

Despite considerable sequence divergence between genera, several geminivirus proteins show reported functional similarities beyond replication. Proteins such as Rep, the CP, TrAP, MP, and betasatellite-encoded βC1 repeatedly target core host machinery (Sunter and Bisaro, 1997; Castillo et al., 2004; Li et al., 2018). These proteins consistently facilitate nuclear localization, transcriptional regulation, RNA silencing suppression, and modulation of host defense responses (Beam and Ascencio-Ibáñez, 2020; Shakir et al., 2023). These observations suggest that despite being distinct lineages, geminiviruses can converge on a limited set of fundamental host pathways. However, the available experimental evidence is uneven across *Geminiviridae* and is strongly concentrated in *Begomovirus*; therefore, the extent of functional convergence across the family remains unresolved.

Our understanding of variable genomic features has undergone a substantial transformation. The historical distinction between OW and NW begomoviruses, particularly the absence of the AV2 gene in most NW viruses, is increasingly discussed in the context of functional convergence rather than simple gene loss (Ha et al., 2008; Nawaz-ul-Rehman and Fauquet, 2009; Crespo-Bellido et al., 2024). The positional homolog AV2 in OW begomoviruses functions as an RNA-silencing suppressor and movement-associated factor (Rojas et al., 2001; Rojas et al., 2005; Rothenstein et al., 2007; Zrachya et al., 2007; Chowda-Reddy et al., 2008). In contrast, RNA-silencing suppression in begomoviruses involves multiple viral proteins rather than a single conserved factor. TrAP/AC2 and AC4 have been demonstrated to suppress RNA silencing in several bipartite begomoviruses, whereas CP and AV2/V2 can also contribute to suppression in particular begomovirus species (Zrachya et al., 2007; Amin et al., 2011; Soitamo et al., 2012; Fondong, 2013; Hanley-Bowdoin et al., 2013; Guerrero et al., 2020). Thus, the absence of AV2 in many NW begomoviruses should not be interpreted as a simple one-to-one replacement by TrAP. Rather, the available evidence suggests distributed functional compensation, in which different viral proteins contribute to functions associated with host defense suppression and virus movement. This pattern illustrates the functional flexibility of begomovirus genomes in adapting to host immune pressures.

DNA satellite molecules provide an important evolutionary perspective on the genomic plasticity and functional diversification of geminivirus-associated systems. Rather than representing a newly recognized role in geminivirus biology, established studies have demonstrated that betasatellites can act as major pathogenicity determinants in several economically important disease complexes. The betasatellite-encoded βC1 protein functions as a versatile pathogenicity hub, suppressing host RNA silencing, disrupting hormone signaling and interfering with host autophagy (Sattar et al., 2013; Zhou, 2013; Luna et al., 2017). In addition to betasatellites, alphasatellites with their nanovirus-derived Rep genes and the recently classified deltasatellites illustrate the diversity of geminivirus-associated satellite molecules and their distinct evolutionary histories. More recent studies have also revealed diverse satellite–helper virus interactions, including associations beyond *Begomovirus*, suggesting that satellite molecules may contribute to the evolutionary and genomic diversification of geminivirus-associated system. However, their broaderbiological and evolutionary significance remains incompletely understood and requires further comparative and functional investigation.

## 6 Advances in understanding geminivirus recombination and its evolutionary implications

Over the last two decades, large-scale genomic analyses have established recombination as a fundamental process in shaping geminivirus diversity and evolution, particularly within the genus *Begomovirus*. After this recognition, recombination is now recognized as a primary driver of genetic diversity, often exceeding point mutation in its contribution to genome diversification. Pivotal studies, particularly those by Lefeuvre and colleagues, have demonstrated extensive recombination among begomoviruses and identified non-random distributions of recombination breakpoints across their genomes (Duffy and Holmes, 2008; Lefeuvre et al., 2009; Lefeuvre et al., 2010). These studies support recombination as an important contributor to begomovirus diversification, although its frequency varies among datasets and lineages.

Recombination is not confined to non-coding or IR regions. A key conceptual advance is the model of modular evolution, which involves the exchange of functional gene modules, such as those encoding CP or Rep proteins, as discrete evolutionary units (Padidam et al., 1999; Amrao et al., 2010). Recombination and genetic exchange have also been reported to transcend conventional taxonomic boundaries (Hernández-Zepeda et al., 2013). For example, evidence of genetic exchange between viruses from distinct genera (e.g., *Mastrevirus* and *Begomovirus*) in the *Nicotiana* species suggests that geminivirus evolution is not exclusivelyy vertical (Khalid et al., 2017). Instead, such evidence supports a highly interactive genetic landscape involving horizontal gene flow between distinct geminivirus genera. Such intergeneric recombination events blur traditional taxonomic boundaries and highlight the genetic mobility in the family *Geminiviridae* (Rocha et al., 2013). However, the frequency and evolutionary significance of intergeneric recombination across the family remain poorly quantified. This provides the basis for modular evolution models, in which coding sequences such as Rep, CP and MP can acquire distinct evolutionary histories through recombination (Padidam et al., 1999; Amrao et al., 2010).

Recent studies have provided the evidence for mosaic evolution of geminiviruses. For example, in *Becurtovirus*, virion-sense genes show curtovirus-like affinities, whereas Rep is more closely related to mastreviruses, while *Citlodavirus* exhibits a begomovirus-like CP but a Rep related to *Becurtovirus* (Fontenele et al., 2018; Zhang et al., 2018). Similarly, *Eragrovirus* combines a mastrevirus-like CP with Rep showing relationships to begomoviruses, curtoviruses, and topocuviruses, together with a distinctive TAAGATTCC replication-origin motif shared with *Becurtovirus* (Varsani et al., 2009; Varsani et al., 2014b). Additional evidence comes from *Topocuvirus*, for which a recombinant origin has been proposed involving nightshade curly top virus as the major parent and beet curly top virus as the minor parent (Sun et al., 2021). Its genome contains features resembling both *begomovirus*/*curtovirus* and *becurtovirus* architectures, further illustrating how recombination can generate novel genomic combinations. *Welwivirus* provides another example of evolutionary incongruence: its Rep proteins cluster with *Capulavirus* and *Topilevirus*, whereas its CP is more closely related to *Mastrevirus*. Although a specific recombination event has not been demonstrated, this discordance is consistent with an ancient recombinant origin followed by extensive sequence divergence (Debat and Bejerman, 2022; Bejerman and Debat, 2023). Collectively, these examples indicate that geminivirus evolution is better represented as a network of interacting genetic modules than as a strictly vertical, tree-like process.

Several well-documented cases provide evidence of recombination in geminiviruses. Tomato yellow leaf curl virus-Israeli strain (TYLCV-IL) and tomato yellow leaf curl Sardinia virus-Spanish strain (TYLCSV-ES) produced a recombinant TYLCV-IS76 in Souss in late 1990s. Emergence of this recombinant coincided with the increasing use of resistance tomato cultivars possessing *Ty-1* gene. The selective advantage helped this recombinant to achieve entire displacement of both parental viruses from Souss (Belabess et al., 2016). Moreover, recombination between co-circulating begomovirus species in geographically distant regions where parental viruses co-existed have showed to generate the same emergent lineage repeatedly. It is evidenced in the independent recurrent emergence of tomato yellow leaf curl Malaga virus in Spain and Portugal and tomato yellow leaf curl Axarquia virus in Spain and Italy (Fiallo-Olivé et al., 2019). In the cotton leaf curl disease (CLCuD) complex, many recombination events are recorded. Recombination between cotton leaf curl Multan virus (CLCuMV) donating its Rep protein and cotton leaf curl Kokhran virus (CLCuKoV) contributing its CP has generated several new viral sequences (Saleem et al., 2016). A notable case is the highly recombinant cotton leaf curl Multan virus-Rajasthan strain that evolved from inter-species recombination involving multiple donors (Biswas et al., 2020). Recently, a novel recombinant virus was found infecting bottle gourd in Kenya. This recombinant was shown as chimera of tomato leaf curl Uganda virus (ToLCUV) and tomato leaf curl Kunene virus (ToLCKunV) (Ringeera et al., 2025).

The recognized role of DNA satellite molecules further expands this genetic landscape. Previously regarded as subgenomic components, DNA satellites, particularly betasatellites, are now recognized as important participants in recombination and genetic diversification. Studies of disease complexes such as the CLCuD complex have revealed frequent recombination among betasatellites (Zubair et al., 2021). A well-characterized case is the emergence of CLCuMB in which Burewala and Shahdadpur strains contain recombinant fragments within the SCR region derived from tomato leaf curl betasatellite (ToLCB) (Zubair et al., 2017). Similarly, cotton leaf curl Lucknow alphasatellites, CLCuMA, ageratum yellow vein Indian alphasatellites and ageratum enation alphasatellites are likely to have evolved through recombination (Mubin et al., 2022). Intra-satellite recombination can generate novel variants that associate with diverse helper begomoviruses, potentially facilitating the spread of pathogenicity-associated satellite functions across different viral backgrounds (Kandito et al., 2021; Biswas et al., 2025).

Inter-satellite recombination has also been documented in geminiviruses. A novel defective satellite molecule (RecSat), found in association with tobacco leaf curl Yunnan virus (TbLCYNV) in infected tobacco plants, was proposed to have evolved through three independent recombination events involving tomato yellow leaf curl China betasatellite, ageratum yellow vein China betasatellite and tobacco curly shoot alphasatellite (Huang et al., 2013). RecSat contained recombinant sequences derived from different satellite types and could be trans-replicated by its helper virus, demonstrating that satellite molecules can undergo genetic exchange while remaining dependent on helper-virus replication. However, the frequency and precise mechanisms of satellite–helper-virus recombination remain insufficiently characterized.

### 6.1 Anthropogenic drivers of geminivirus dissemination and emergence

More critically, beyond genome-intrinsic mechanisms, human activity is directly connected to the global spread and subsequent evolution of geminiviruses. A representative case is the global spread of TYLCV through multiple independent introductions from the Middle East into the Americas, Asia, and Europe, which has been linked to international trade of infected seedlings and plant material (Lefeuvre et al., 2010). Similar patterns have been reported of other economically important begomoviruses, such as the rapid expansion of ToLCNDV from the Indian subcontinent to Southeast Asia, the Mediterranean region, and West Africa, likely facilitated by the movement of infected cucurbit and tomato plant materials (Iqbal et al., 2025). Likewise, the regional dissemination of ToLCPalV from northwestern India first to Pakistan and then westward to Iran and other Middle Eastern countries (Sattar et al., 2025; Sattar et al., 2026), and the introduction of watermelon chlorotic stunt virus (WmCSV) from the Middle East into North Africa and the Mediterranean (Iqbal, 2025; Sattar et al., 2025). All these cases are correlated with regional and international agricultural trade networks. Consequently, geminivirus evolution is now viewed as a dynamic, multi-layered process driven by both intergeneric and intrageneric recombination. These examples illustrate patterns of human-associated geminiviruses spread rather than newly emerging events. The example of TYLCV represents a well-documented case of long-distance movement through infected planting material; ToLCPalV represents recent cross border dissemination across South and West Asia; and WmCSV represents an unknown intercontinental introduction, where the mechanism of movement remains unresolved. Collectively, these cases demonstrate that agricultural trade and movement of infected plant material can facilitate both geographic expansion and exposure of geminiviruses to new environmental settings. Climate change may further modify the evolutionary dynamics of geminiviruses by altering the conditions required for their transmission. The experimental data on TYLCV suggests that heat stress can increase the accumulation of virus in *Bemisia tabaci* and enhances its transmission efficiency (Wang et al., 2022).

The agricultural production systems may indirectly influence the geminiviruses evolution. Monoculture, repeated cropping pattern, and change in crop phenology can increase the temporal availability of susceptible hosts. Whereas intensive insecticide use can impact the vector population dynamics and impose selection on vector population. These factors may influence viral selection, recombination and opportunities for co-infection. However, direct evidence that individual agricultural practices such as monoculture, irrigation or pesticide application drive specific evolution in geminiviruses genomes remains limited.

### 6.2 Evolutionary forces and limitations of recombination analysis

Earlier studies conducted on cassava mosaic viruses showed that different African and Indian whitefly populations transmitted different cassava mosaic viruses with markedly varying efficiencies (Liu et al., 1997; Maruthi et al., 2002). Research on bean golden mosaic virus showed that molecules mutant for coat protein gene could still establish systemic infection when DNA-B was supplied. However, such CP mutants were not able to be transmitted by whiteflies. The experiments conducted by Liu et al., demonstrated that efficient whitefly transmission of African cassava mosaic geminiviruses required sequences both from DNA-A and DNA-B. They constructed pseudo-recombinants between transmissible and non-transmissible ACMV isolates. Indeed, pseudo-recombinants could not restore transmission indicating the fact that determinants on both genomic components contribute to transmission. It was further found that virus accumulation within the insect was dependent on DNA-B component. Whereas, virus transmission in the plants depended on DNA-A component, with the coat proteins being essential for acquisition of virus particle (Liu et al., 1997). Further work on Abutilon mosaic virus (AbMV) gave direct evidence that DNA-B alone did not explain the transmission defect (Höhnle et al., 2001). Instead, a three amino-acid change in CP was sufficient to convert a non-transmissible AbMV isolate into a transmissible virus (Höhnle et al., 2001). Recent studies on squash leaf curl China virus (SLCCNV) revealed that a single residue mutation in coat protein gene did not alter the fitness of the virus but significantly affected transmission characteristics by different *B. tabaci* species (Pan et al., 2020). In consensus with all these studies, it can be concluded that for bipartite begomoviruses, there is a central role for the DNA-A-encoded coat protein in whitefly transmission, whereas DNA-B primarily contributes their movement functions. In monopartite begomoviruses, these functions are integrated within a single DNA-A-like genome, making transmission-related determinants part of the same genomic unit. The situation becomes more modular for satellite-associated begomoviruses. As betasatellites lack their own coat protein and depend on helper viruses for vector transmission. Experimental transmission of Cotton leaf curl Multan betasatellite in association with a helper begomovirus, and subsequent demonstration of vector-mediated transfer of a betasatellite between helper viruses, illustrate how selection and compatibility can operate across interacting genomic units (Tabein et al., 2013).

Viral evolution is highly dynamic and is driven by mutation, recombination and pseudo-recombination (re-assortment), with natural selection, genetic drift and gene flow subsequently shaping viral genomic structure (LaTourrette and Garcia-Ruiz, 2022). Within this evolutionary framework, RNA viruses have mutation rates of 10^−6^ to 10^−4^ substitutions per nucleotide per cell infection compared with 10^−8^ to 10^−6^ substitutions per nucleotide per cell infection for DNA viruses (Sanjuán and Domingo-Calap, 2016). However, these ranges are not specific to geminiviruses and this distinction becomes less pronounced in ssDNA viruses and the viruses with small genomes as they show higher mutation rates (Sanjuán et al., 2010; Sanjuán and Domingo-Calap, 2016). The rolling circle and recombination-dependent mechanism of replication creates opportunities for both sequence variation and genomic rearrangements. Most importantly, recombination is unevenly distributed across their genomes. A further geminivirus-specific feature is pseudo-recombination, where a complete genomic component instead of individual sequence segment is exchanged (Gilbertson et al., 1993). Geminiviruses provide an important example of this pattern, with studies reporting high levels of genetic variation and rapid evolutionary change. For-example, substitution rates measured in several begomoviruses have been estimated at approximately 10^−4^ -10^−3^ substitutions per site per year (Duffy and Holmes, 2008; Duffy and Holmes, 2009). Beyond the rate of genetic change, viral populations can also differ in their evolutionary dynamics. In an evolving viral population, viruses can occupy an evolutionary continuum between two conceptual evolutionary states. Stationary states occur near equilibrium and involve relatively slow evolutionary change, whereas transient states are far from equilibrium and characterized by rapid evolutionary change. Geminiviruses exhibit several characteristics consistent with transient evolutionary dynamics. These characteristics include relatively rapid evolutionary rates, substantial genetic variation, and frequent recombination (Padidam et al., 1999; Duffy and Holmes, 2008; Duffy and Holmes, 2009). Although the available evidence does not support assigning all geminiviruses uniformly to a transient state. The trajectory of an evolving viral population is dictated by selection dynamics. Purifying selection favors stationary dynamics whereas directional or diversifying selection favors transient dynamics (de Oliveira et al., 2012). Moreover, viral populations are subject to selection constraints imposed by vector determinants, host-associated selection and environmental factors (LaTourrette and Garcia-Ruiz, 2022). Furthermore, these selective forces may be counterbalanced by bottlenecks imposed by vector transmission, environmental conditions, presence of susceptible hosts within a population and antiviral defense (Moury et al., 2017; Garcia-Ruiz, 2018; LaTourrette and Garcia-Ruiz, 2022). At the ecological level, the transition of geminiviruses between reservoir hosts and seasonal crops has been proposed as a stringent bottleneck. As it can reduce the viral diversity entering the crop population (Nawaz-ul-Rehman et al., 2012). However, this interpretation is still qualitative, because the number of viral genotypes successfully transmitted through this transition have not been experimentally proven. These bottlenecks can stochastically reduce the genetic diversity of viral populations (Ali and Roossinck, 2008). Thus, geminiviral evolution is governed by a combination of mutation, recombination, selection, genetic drift, population structure, gene flow and transmission-associated demographic processes rather than recombination alone.

Recombination analysis of geminiviruses relies largely on computational tools, but their results can be influenced by several methodological limitations. Commonly used methods include RDP, GENECONV, BootScan, MaxChi, Chimaera, SiScan, and 3Seq, which differ in their assumptions and detection sensitivity (Crespo-Bellido et al., 2025). At the input level, factors such as uneven sampling, variations in sample size, sequence diversity and recombination frequencies can substantially affect the accuracy of recombination detection programs (Posada and Crandall, 2001; Martin et al., 2011; Jaya et al., 2023). These data challenges are further compounded by limited scalability and resolution of recombination detection programs (Jaya et al., 2023). Recombination can also generate complex mosaic genomes that complicate phylogenetic inference because different regions may have distinct evolutionary histories (Schierup and Hein, 2000; Posada and Crandall, 2001). Repeated recombination events can further complicate the genomic mosaic structures, making recombination signals difficult to resolve reliably (Jaya et al., 2023). Therefore, no single algorithm can be considered universally reliable, and a multi-method approach combined with phylogenetic validation provides greater confidence in identifying genuine recombination events (Crespo-Bellido et al., 2025).

Alignment quality presents another major challenge in recombination analysis. Traditional alignment-based tools can be computationally expensive and time-consuming and may scale poorly with increasing dataset size, whereas newer alignment-free methods still require further optimization (Jaya et al., 2023). Finally, methodological differences among recombination detection algorithms, including distance-based, phylogenetic, compatibility or substitution distribution approaches, can result in substantial differences in performance. Different methods have distinct statistical power, false-positive rates and resolution for identifying recombinant sequences and inferring their potential parental relationships (Posada and Crandall, 2001; Jaya et al., 2023).

## 7 Functional convergence and adaptive divergence in host pathway targeting by geminiviruses

Although different genera of geminiviruses are phylogenetically and genomically diverse, functional studies across several genera reveal recurrent targeting of essential host processes, including viral replication, movement and suppression of host defense system. Nevertheless, the evidences are highly uneven across the family. Begomoviruses account for a substantial proportion of the mechanistic studies, reflecting both its large species diversity and its extensive experimental characterization. While *Mastervirus* and *Curtovirus* have been investigated for selected host interactions and several other genera remain poorly characterized functionally. Therefore, the recurring involvement of these pathways is interpreted as evidence of partial functional convergence rather than uniformly demonstrated convergence across *Geminiviridae*.

A fundamental requirement for geminivirus replication is the induction of a S-phase-like environment in differentiated plant cells. To achieve this, geminiviruses inhibit the key plant cell-cycle regulator, retinoblastoma-related protein (RBR) (Gutzat et al., 2012; Figure 3A). In plants, RBR regulates gene expression by binding E2F transcription factors. Geminiviruses inhibit this binding of RBR to E2F through their Rep, REn and Rep A proteins (Kong et al., 2000; Desvoyes et al., 2006; Hanley-Bowdoin et al., 2013). The Rep-RBR interaction displaces E2F transcription factors, which in turn activate host DNA-synthesis genes. This transcriptional reprogramming recruits essential host replication machinery as Rep interacts with replication factor C and replication protein A to facilitate proliferating cell nuclear antigen (PCNA) loading and support viral replication. Moreover, REn also contributes by interacting with PCNA (Luque et al., 2002; Castillo et al., 2003; Bagewadi et al., 2004; Singh et al., 2007). Geminivirus genera also differ in their mechanisms of RBR interaction: mastreviruses use the LXCXE RBR-binding motif in Rep, whereas begomoviruses and curtoviruses use distinct Rep motifs to bind RBR (Liu et al., 1999; Arguello-Astorga et al., 2004; Lageix et al., 2007; Hanley-Bowdoin et al., 2013).

**Figure 3:**
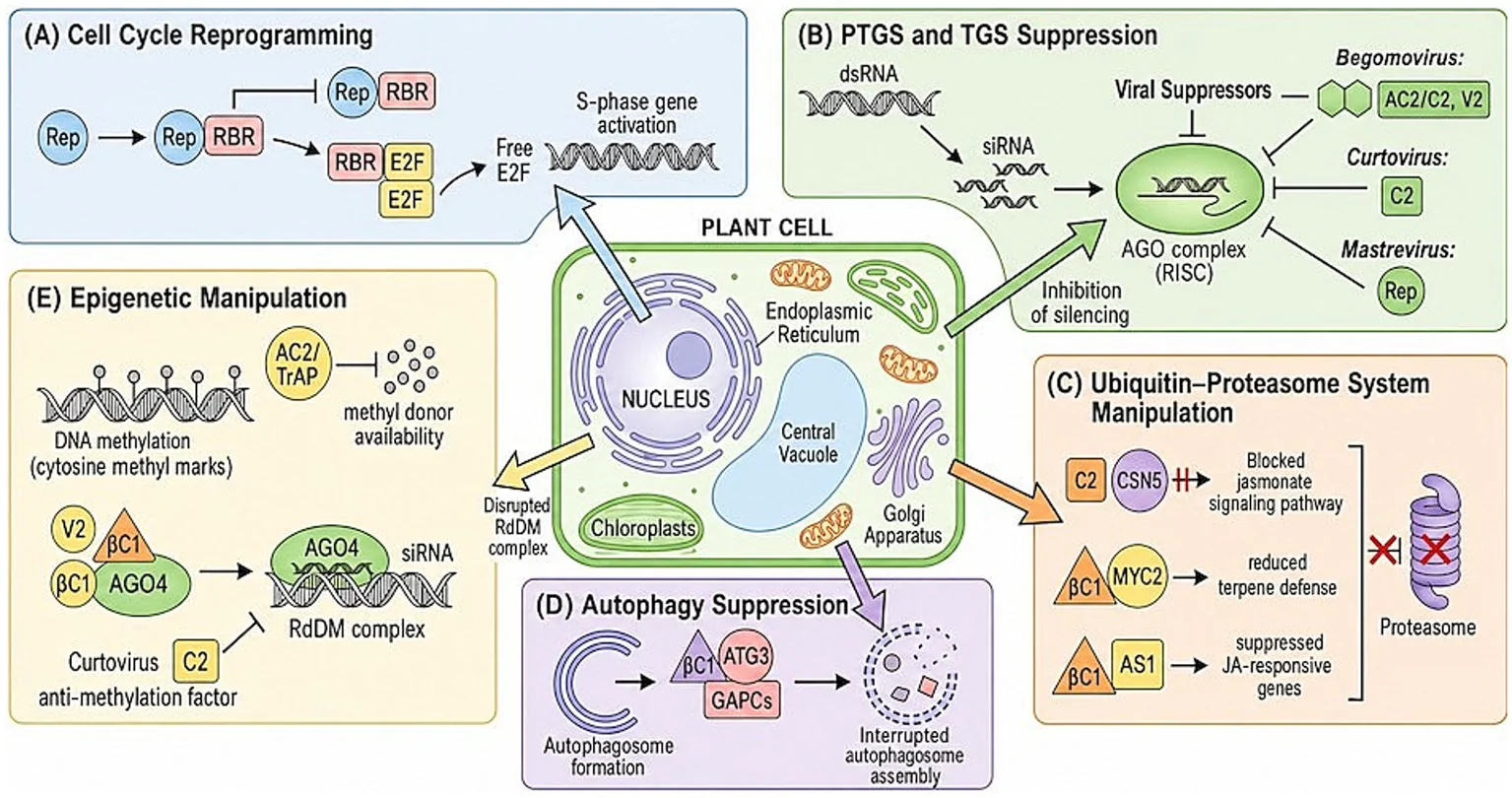
Reported functional similarities among geminivirus proteins beyond replication. **(A)** Manipulation of host cell-cycle regulation through RBR-E2F interaction; **(B)** Suppression of RNA silencing by viral suppressors, including post-transcriptional gene silencing (PTGS); **(C)** Interference with the ubiquitin-proteasome system and hormone signaling pathways, **(D)** Modulation of autophagy pathways by viral proteins; and **(E)** Interference with transcriptional gene silencing (TGS) through disruption of the RNA-directed DNA methylation (RdDM) machinery. The figure summarizes experimentally reported interactions with major host pathways and represents a qualitative synthesis rather than a quantitative comparison among geminivirus genera. Available evidence is uneven across *Geminiviridae* and is particularly concentrated in *Begomovirus*.

RNA interference (RNAi)-based approaches have been widely exploited to engineer resistance to geminiviruses by targeting essential viral genes such as Rep and CP (Ye et al., 2014; Singh et al., 2015). Expression of hairpin RNA constructs or microRNAs in transgenic plants has shown effective suppression of viral replication and symptom development (Ali et al., 2013; Al-Roshdi et al., 2023). Additionally, virus-induced gene silencing (VIGS) systems further enhance RNAi-mediated defense against geminiviruses (Peele et al., 2001; Lentz et al., 2018; Watanabe et al., 2021). As a counter-defense strategy, most geminiviruses encode one or more RNA-silencing suppressors (RSSs), that target post-transcriptional gene silencing (PTGS), transcriptional gene silencing (TGS), or both (Figure 3B; Hanley-Bowdoin et al., 2013; Kumar, 2019; Beam and Ascencio-Ibáñez, 2020; Gong et al., 2021; Veluthambi and Sunitha, 2021; Zhang et al., 2023). However, the viral proteins involved are largely non-orthologous, indicating convergent evolution rather than shared ancestry. For example, AC2/C2 in begomoviruses and C2 in curtoviruses are positional homologs with partial functional similarity because both can suppress TGS but AC2/C2 in begomoviruses uses a C-terminal acidic activation domain for transcription-dependent silencing suppression whereas C2 in curtovirus lacks this domain and employs activation-domain-independent pathways of suppression (Guerrero et al., 2020; Luna and Lozano-Durán, 2020; Veluthambi and Sunitha, 2021). Moreover, begomoviruses employ multiple RSSs: AC2/TrAP inhibits adenosine kinase and methyl cycle enzymes (Bisaro, 2006; Veluthambi and Sunitha, 2021), AC4 sequesters small RNAs and interferes with Argonaute 4 (AGO4) function (Vinutha et al., 2018) and V2 binds small interfering RNAs (siRNAs) and suppresses RDR6–SGS3 amplification (Glick et al., 2008; Luna et al., 2017; Zhang et al., 2023). In contrast, curtoviruses primarily depend on C2 protein, which acts as a transcriptional activator and participates in suppressing methylation pathways (Veluthambi and Sunitha, 2021). Mastreviruses frequently employ Rep as a multifunctional RSS to effectively link replication and counter-defense (Wang et al., 2014; Veluthambi and Sunitha, 2021). Additionally, in monopartite begomovirus complexes, the betasatellite-encoded βC1 acts as an established, multifunctional RSS that promotes SGS3 degradation, inhibits RDR6 activity, and interferes with AGO4-mediated methylation, thus suppressing both PTGS and TGS (Li et al., 2014a; Kamal et al., 2019; Zhang et al., 2023). Beyond these activities, βC1 is also involved in destabilizing key silencing components by recruiting host ubiquitin ligases (Eini et al., 2009; Yang et al., 2011b; Hanley-Bowdoin et al., 2013). These findings illustrate how satellite acquisition can enhance viral counter-defense capacity.

In addition to replication and silencing suppression, geminiviruses exhibit several lineage-specific adaptations that manipulate additional host systems such as the ubiquitin–proteasome system (UPS), hormone signaling, autophagy, and epigenetic regulation, reflecting diverse evolutionary trajectories. Begomoviruses and curtoviruses, for example, frequently manipulate the ubiquitin–proteasome system to modulate hormonal signaling and degrade host defense proteins (Lozano-Durán et al., 2011; Hanley-Bowdoin et al., 2013; Zhang et al., 2023). A well-known example is the C2 protein encoded by certain TYLCV isolates, which interacts with the COP9 signalosome subunit 5 (CSN5) to interfere with jasmonate signaling by inhibiting the ubiquitin ligase activity of SKP1–Cullin1–F-box (SCF) complex (Figure 3C; Lozano-Durán et al., 2011). Moreover, the alteration of plant hormone signaling pathways represents a recurring strategy used by begomovirus-betasatellite complexes to facilitate infection and symptom development (Yang et al., 2008; Eini et al., 2009; Zhang et al., 2023). Similarly, βC1 interacts with factors such as basic helix–loop–helix transcription factor MYC2 and ASYMMETRIC LEAVES 1 (AS1) to suppress terpene-mediated and jasmonate-responsive defenses resulting in improved vector performance and altered developmental gene expression (Yang et al., 2008; Li et al., 2014b).

Autophagy has recently emerged as a critical host defense mechanism that targets viral proteins for degradation. Begomovirus-betasatellite complexes actively counteract this pathway through the βC1 protein (Figure 3D). βC1 interacts with autophagy-related protein 3 (ATG3) and glyceraldehyde-3-phosphate dehydrogenases (GAPCs), disrupting autophagosome formation and preserving viral proteins. Autophagy modulation appears less prominent in other geminivirus genera which suggests a begomovirus-specific molecular arms race driven by complex host interactions and satellite acquisition (Ismayil et al., 2020a; Ismayil et al., 2020b; Zhou et al., 2021).

Epigenetic manipulations represent another layer of host–virus interactions (Figure 3E). Plants impose a strong barrier to geminivirus replication through TGS mediated by RNA-directed DNA methylation (RdDM). Begomoviruses have evolved sophisticated countermeasures: to reduce methyl-donor availability, AC2/TrAP inhibits S-adenosyl methionine synthetase (SAMS) (Wang et al., 2005; Raja et al., 2008; Yang et al., 2011b; Hanley-Bowdoin et al., 2013) and Rep, with the assistance of C4, downregulates DNA methyltransferase 1 (MET1) and chromomethylase 3 (CMT3) (Hanley-Bowdoin et al., 2013; Rodríguez-Negrete et al., 2013) while V2 and βC1 directly interact with AGO4 to disrupt RdDM complex assembly (Zhang et al., 2023). Although curtovirus C2 exhibits mild antimethylation activity (Zhang et al., 2011), mastreviruses appear to lack dedicated epigenetic suppressors, possibly relying on transient infection strategies to avoid silencing (Wang et al., 2014; Veluthambi and Sunitha, 2021). These host manipulation strategies represent key adaptive traits that enhance viral fitness. The recurrence of these mechanisms across different genera suggests that they are important to successful infection. At the same time, variation in how these strategies are deployed points to lineage-specific evolutionary adaptations shaped by host interactions and ecological factors.

Thus, there are two major evolutionary dynamics shape the geminivirus–host interactions landscape. The first is conserved targeting of essential host pathways such as cell cycle regulation and RNA silencing (Hanley-Bowdoin et al., 2013). A second pattern is lineage-specific diversification that include ubiquitin–proteasome system manipulation, hormone signaling, autophagy evasion, and epigenetic control (Ascencio-Ibáñez et al., 2008; Hanley-Bowdoin et al., 2013; Beam and Ascencio-Ibáñez, 2020; Zhang et al., 2023). Their evolutionary significance lies in the different viral proteins and molecular mechanisms through which divergent geminivirus lineages manipulate related host processes. However, most of the experimental evidences are characterized on begomoviruses and a smaller number of other genera, consequently, their distribution across *Geminiviridae* remains incompletely resolved. DNA satellite acquisition has further triggered divergence, especially in begomoviruses, by introducing new virulence factors (such as βC1) that broaden the functional capacity of geminiviruses and enhance their pathogenic potential. Certain genera, such as *Mastrevirus*, possess compact genomes and narrow host ranges. These viruses rely on their multifunctional proteins such as Rep, which performs both replication and counter-defense roles (Hanley-Bowdoin et al., 2013; Wang et al., 2014; Beam and Ascencio-Ibáñez, 2020; Veluthambi and Sunitha, 2021; Zhang et al., 2023). This preference for genetic economy over the acquisition of specialized, modular virulence factors may reflect an evolutionary strategy that avoids the genomic expansion required for more complex, multiprotein systems. Future research integrating structural biology, comparative genomics, and host–virus interaction networks will further elucidate how functional convergence and adaptive divergence continue to shape geminivirus evolution.

Advancing our understanding of geminivirus genome architecture and host defense modulation, next generation sequencing (NGS) has become a pivotal tool for analyzing the diversity, evolution, and epidemiology of geminiviruses. Collectively, NGS-based studies have transformed geminivirus research from simple virus detection toward population-level evolutionary analysis, enabling high-resolution investigation of recombination, diversification, host adaptation, and virus–satellite interactions.

## 8 Global patterns of geminivirus distribution and diversification

To examine geographic patterns of geminivirus richness, virus and DNA-satellite records were retrieved from NCBI Virus^2^ and mapped to ICTV taxonomy (ICTV, 2025; ICTV, 2026) according to the proposed geminivirus diversification framework of Nawaz-ul-Rehman and Fauquet (2009). These records were assigned to eight predefined analytical regions: Australia, Japan, China, the Indian subcontinent, Africa, the Mediterranean-European region, Central America and South America. For this geographic analysis, Indian subcontinent center includes Bangladesh, Bhutan, India, Indonesia, Iran, Laos, Malaysia, Myanmar, Nepal, Oman, Pakistan, Sri Lanka, Thailand, and Cambodia. In comparison to the dataset available in 2009, the current analysis comprises of substantially updated ICTV taxonomy records. Therefore these regions retained as predefined analytical units to facilitate comparison, and are not interpreted as fixed biological boundaries. Species and genus richness were calculated separately for geminiviruses, alphasatellites and betasatellites (Figure 4; Supplementary Data Sheets S1–S4).

**Figure 4:**
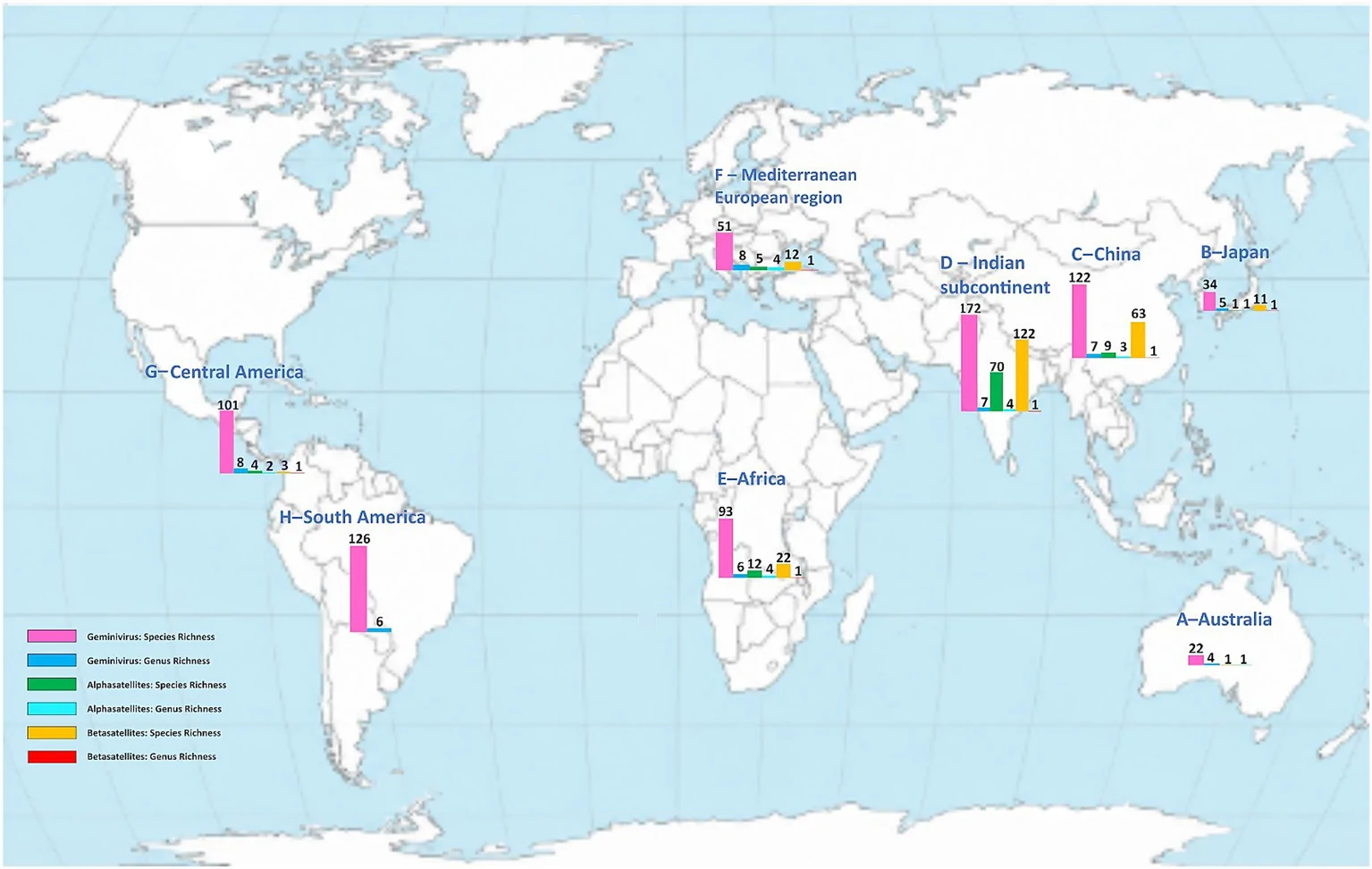
Global distribution patterns of geminiviruses and their associated DNA-satellites across eight major diversification regions. A– Australia, B – Japan, C – China, D – Indian subcontinent, E – Africa, F – Mediterranean-European region, G – Central America, H – South America. To calculate the geographically documented taxonomic composition of geminiviruses and their associated satellites, data was retrieved from NCBI Virus (https://www.ncbi.nlm.nih.gov/labs/virus/vssi/), and their identities were mapped to the ICTV’s MSL41 and Virus Metadata Resource (VMR) current version (ICTV, 2025; ICTV, 2026). In this mapping, isolates and strains were treated as records belonging to the same taxon. Duplicate records were removed and only distinct records were retained for analysis. Thus, number of NCBI records representation is distinct from the taxonomic richness. Most importantly, when a species was represented by records from multiple countries within the same predefined geographic region, that species was counted only once within that region when calculating regional species richness. However, the global species richness was calculated from the 549 unique species across the complete dataset. The regional richness values were calculated independently, and therefore are not expected to sum to the global total.

Geminivirus species and genus richness varied substantially across the eight surveyed regions, with the Indian sub-continent recording the highest richness (172 species and 7 genera). This was followed by South America (126 species from 6 genera), China (122 species from 7 genera), and Centra America (101 species and 8 genera). Intermediate richness values were found in Africa (93 species and 6 genera) and the Mediterranean-European region (51 species and 8 genera). The lowest richness values were recorded in Japan (34 species from 5 genera) and Australia (22 species from 4 genera). The Indian subcontinent has been identified as a principal center of origin and diversification of geminiviruses, consistent with the findings of Nawaz-ul-Rehman and Fauquet (2009).

Notably, high genus richness does not necessarily correspond to a high species count in a region. For example, Mediterranean-European region and Central America each host 8 genera, but their species richness is considerably lower than that of the Indian sub-continent. The difference of genus and species richness indicates that these two measures capture different levels of geminivirus diversity. A region may therefore accumulate representatives of multiple divergent lineages without extensive species level richness within a lineage, whereas another region may support higher diversification with a smaller number of established lineages. However, these mechanisms cannot be distinguished directly from the present descriptive analysis. The differences in sampling and sequence availability among regions may also contribute to the observed pattern.

Several factors may contribute to species and genus richness of geminiviruses. These include the worldwide expansion of geminivirus diversity (Kraberger et al., 2013; Malathi et al., 2017; Varma and Singh, 2024), rapid viral evolution (Mishra et al., 2020), frequent intra- and interspecific recombination (Venkataravanappa et al., 2011; Biswas et al., 2025), the spread of invasive *B. tabaci* cryptic species or biotypes with high transmission efficiency (Esterhuizen et al., 2013), frequent mixed infections in host plants (Kumar, 2019; Mishra et al., 2020) and global trade of infected plant material (Esterhuizen et al., 2012). The introduction of exotic crops may also contribute to geminivirus spread, as illustrated by the detection of East African cassava mosaic virus (EACMV) and ACMV in Angola (Lava Kumar et al., 2009). Host-range expansion may also contribute, as illustrated by reports of cotton leaf curl Gezira virus (CLCuGeV) strains, originally associatercad with cotton in Egypt and Sudan, infecting beans in the southwest Indian Ocean (SWIO) region (Lefeuvre et al., 2007). The persistence of alternative hosts and the use of advanced detection and diagnostic tools may also contribute to apparent increases in documented records. For example, rolling circle amplification (RCA) combined with Oxford Nanopore sequencing detected pepper yellow vein Mali virus (PepYVMLV) and cotton leaf curl Gezira alphasatellite (CLCuGeA) in the sweet potato virome in Burkina Faso for the first time (Name et al., 2025).

Betasatellites are predominantly associated with OW begomoviruses, whereas natural betasatellite associations are rarely documented among NW begomoviruses. Alphasatellites and deltasatellites, however, have been documented in the OW. The Indian-subcontinent had the highest satellite richness, with 7 alphasatellites species from 4 genera and 122 betasatellites species from 1 genus, consistent with published reports (Venkataravanappa et al., 2011; Kumar et al., 2015; Kumar et al., 2017). This was followed by China (9 alphasatellites species from 3 genera and 63 betasatellites species from 1 genus), Africa (12 alphasatellites species from 4 genera and 22 betasatellites species from 1 genus), Mediterranean-European region (5 alphasatellites species from 4 genera and 12 betasatellites species from 1 genus), and Japan (1 alphasatellites species from 1 genus and 11 betasatellites species from 1 genus). In contrast, satellites were essentially absent from the NW (Briddon et al., 2003; Van Brunschot et al., 2010).

## 9 Proposed models and theories of geminivirus evolution

The evolutionary origins of *Geminiviridae* family remain incompletely understood partly because of their chimeric biological characteristics. Despite having circular ssDNA genomes, geminiviruses have evolutionary rates comparable to RNA viruses. They replicate through RCR mechanism, which is a feature typical of many plasmids (Duffy and Holmes, 2008; Kazlauskas et al., 2019). Furthermore, structural analyses of geminiviruses reveal that their CPs have more similarity to those of icosahedral RNA viruses than with other DNA viruses (Krupovic et al., 2009). Collectively, these mixed characteristics have led to several hypotheses regarding the evolutionary origins of the replication and structural components that gave rise to modern geminiviruses (Figure 5A). Some of these hypotheses are partially conflicting. They are also summarized in Supplementary Table S2.

**Figure 5:**
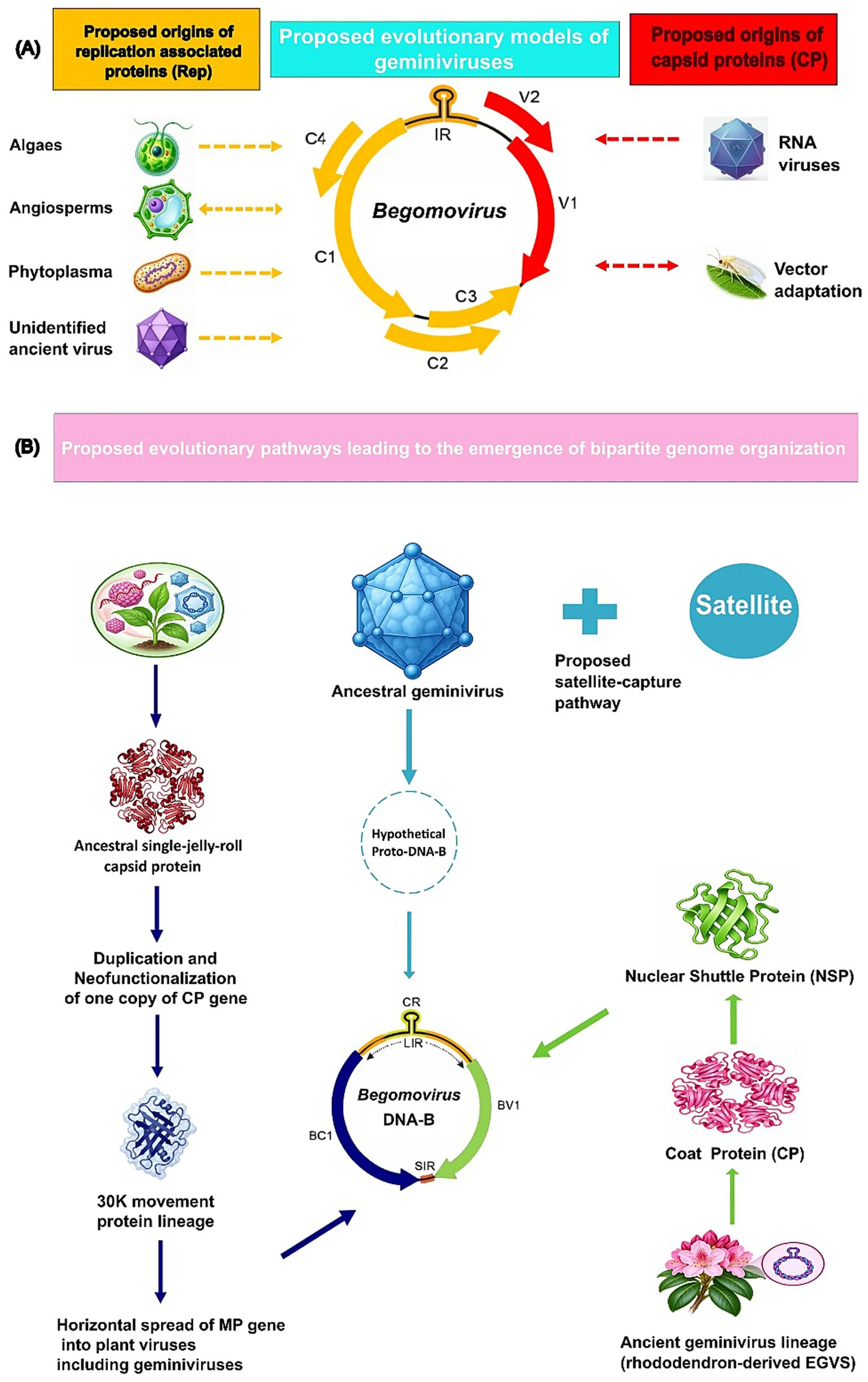
Proposed evolutionary models of geminiviruses. **(A)** Hypothesized evolutionary origins of geminivirus replication-associated protein (Rep) from algae (Nawaz-ul-Rehman and Fauquet, 2009; Nash et al., 2011), phytoplasma (Nishigawa et al., 2001; Krupovic et al., 2009), and an unidentified ancient virus (Torres-Herrera et al., 2019), and of capsid proteins (CP) from RNA viruses, such as satellite tobacco necrosis virus (Krupovic et al., 2009). Proposed reciprocal evolutionary association (co-adaptation) between insect vectors and geminivirus CP (Lefeuvre et al., 2019; Dolja et al., 2020) and between angiosperms and geminivirus Rep,are also shown (Sharma et al., 2020). Single-headed dashed arrows indicate possible evolutionary transitions, whereas double-headed dashed arrows denote reciprocal evolutionary associations. Solid arrows indicate proposed evolutionary relationships supported by published comparative or structural evidence, whereas dashed arrows indicate hypothetical or unresolved relationships. **(B)** Proposed evolutionary pathways associated with the emergence of bipartite genome organization and the distinct evolutionary origins of DNA-B encoded movement-associated proteins. The satellite-capture model proposes a hypothetical intermediate, termed “Proto-DNA-B,” between an ancestral geminivirus and the DNA-B component (Nawaz-ul-Rehman and Fauquet, 2009). The proposed model suggests that acquisition of CP contributed to the evolution of functions required for systemic movement and vector mediated transmission. The model further proposes that movement associated proteins later on acquired distinct evolutionary histories, contributing to the emergence of bipartite genome organization. A separate evolutionary pathway shows the proposed origin of the nuclear shuttle protein (NSP) from the CP of an ancient geminivirus lineage represented by rhododendron-derived endogenous geminivirus-like elements (Cárdenas-Conejo et al., 2025). Another pathway illustrates the proposed origin of 30 K movement protein (MP) through ancestral single-jelly-roll (SJR) CP duplication followed by neofunctionalization and subsequent horizontal spread among plant viruses, including geminiviruses (Butkovic et al., 2023). In geminiviruses, this movement-protein pathway is represented by the BC1/MP component of DNA-B and does not imply a common origin of BV1/NSP. Together, these pathways support a modular evolutionary model in which the two DNA-B encoded movement-associated proteins have distinct evolutionary origins. “Proto-DNA-B” is a hypothetical intermediate used here to illustrate the proposed satellite-capture model and is not a directly observed genomic entity.

Earlier models proposed that geminiviruses may have originated from extra-chromosomal DNAs (EcDNAs) of phytoplasmas (Nishigawa et al., 2001; Krupovic et al., 2009). These models hypothesized that a phytoplasmal plasmid acquired a CP gene from a co-infecting plant RNA virus. This event was proposed to have given rise to a primordial geminivirus. Support for these models came from similarity between the Rep protein of geminiviruses and those encoded by phytoplasmas (Duffy and Holmes, 2008; Krupovic et al., 2009). The phytoplasma and plasmid associated origin of geminiviruses replication hypothesis proposes that ancestral replication systems was derived from rolling circle replicating plasmid-like elements. However, the chimeric architecture of geminiviruses suggests the possibility that different genomic modules were acquired from different evolutionary sources. Additionally, CPs of satellite tobacco necrosis virus (STNV) and the geminivirus have been reported to share structural similarity (Jones and Liljas, 1984; Krupovic et al., 2009). However, the phytoplasma-origin hypothesis was challenged by subsequent phylogenetic analyses. These analyses suggested that phytoplasmas may have acquired Rep-like sequences from an ancient geminivirus via horizontal gene transfer rather than the reverse. This interpretation implies that geminiviruses and phytoplasmas may have evolved independently (Saccardo et al., 2011). Several studies have supported this idea that the Rep-like proteins present in phytoplasmal EcDNAs was derived from a geminivirus ancestor rather than from the phytoplasmas themselves. Metagenomic studies based on marine ecosystems challenged the phytoplasma-origin model as they suggested an older evolutionary history of these ssDNA viruses in the marine environments (Kim et al., 2008; Ng et al., 2009; Rosario et al., 2009; Saccardo et al., 2011).

Attention was then shifted to other evolutionary sources of Rep. The discovery of a conserved Geminivirus Rep Sequence (GRS) motif and algal plasmids provided support for the plasmid-to-virus transition hypothesis. This model postulated that geminiviruses have likely evolved from eukaryotic CRESS-DNA plasmids by acquiring a CP gene when infecting an algal or any other protist host (Rojas et al., 2005; Nawaz-ul-Rehman and Fauquet, 2009; Nash et al., 2011). Acquisition of the CP gene may have contributed to encapsidation, cell-to-cell movement, and vector-mediated transmission.

Later, the role of recombination with ancient or unknown viral lineages was also focused in the evolution of geminiviruses (Torres-Herrera et al., 2019). Evidence is found in the evolution of squash leaf curl virus (SLCuV) lineage that possibly involved a recombination event between a NW begomovirus and an unidentified virus. This recombination event may have contributed novel sequences to the Rep and AC4 genes of SLCuV (Torres-Herrera et al., 2019).

The identification of endogenous geminivirus-like elements (EGVs) integrated into numerous angiosperm genomes (*Camellia sinensis*) has provided evidence of a complex coevolutionary history (Sharma et al., 2020). These genomic fossils have shown that geminiviruses or their close relatives have been infecting plants for millions of years. Some EGVs have also retained transcriptional activity. This suggested that plants may have potentially co-opted these EGVs for their development or antiviral defense (Rojas et al., 2005; Sharma et al., 2020).

The origin of the CP remains another major question in geminivirus evolution. Phylogenetic evidence has suggested that geminiviral CPs may share a closer evolutionary relationship with proteins from their insect vectors than with proteins from plant hosts. This relationship has been interpreted as evidence of a long-term virus–vector coevolution in which geminivirus CPs may have evolved under selective pressure to optimize interaction with insect tissues for transmission (Lefeuvre et al., 2019; Dolja et al., 2020).

The acquisition of CP was likely a critical evolutionary step because it may have enabled more than genome encapsidation. It may also have influenced structural and functional diversification within the *Geminiviridae* family. This acquisition event, whether from an insect host (Lefeuvre et al., 2019), an ancestral plant RNA virus (Krupovic et al., 2009) or via reverse transcription of a viral mRNA (Kazlauskas et al., 2019) may have provided the genetic basis for virion assembly and environmental stability. Comparative structural studies (Krupovic et al., 2009; Hesketh et al., 2018) revealed evolutionary convergence between the structural motifs of geminiviruses and those of the small RNA viruses such as STNV. This architectural similarity likely imposed strong selective pressure on these viruses to be efficiently packaged to interact with insect vector receptors for transmission and to withstand extracellular conditions.

The evolution of bipartite geminiviruses and their associated DNA satellites further complicates models of geminivirus evolution. Several hypotheses have been proposed for the origin of the DNA-B genomic component. The satellite-capture model suggests that the DNA-B component originated when an ancestral geminivirus captured a satellite DNA molecule (Nawaz-ul-Rehman and Fauquet, 2009). Separately, Cárdenas-Conejo et al. (2025) recently proposed that DNA-B encoded NSP is evolved from the CP of an ancient geminivirus lineage distinct from modern begomoviruses. They identified rhododendron-derived endogenous geminivirus-like elements as evidence supporting their proposed ancient lineage (Cárdenas-Conejo et al., 2025). In contrast, Butkovic et al. (2023) proposed that the MP originated from single jelly-roll CPs (SJR CP) of plant infecting viruses. Duplication of this SJR CP gene was followed by neofunctionalization of one CP copy into 30 K MPs. These MPs were horizontally transferred to many viruses including geminiviruses (Butkovic et al., 2023). We have hypothesized a modular evolutionary model on the basis of these previously proposed evolutionary scenarios (Figure 5B). As per our proposed evolutionary model, the two DNA-B encoded movement associated proteins have distinct evolutionary origins, as also suggested by (Cárdenas-Conejo et al., 2025).

Comparative studies of DNA-A and DNA-B evolution have shown that divergent evolutionary trajectories for these components in OW viruses. DNA-B appears to display higher genetic diversity and more frequent pseudo-recombination events. Conversely, NW viruses appear to show more consistent coevolution between DNA-A and DNA-B, which results in a more stable evolutionary link between the two components. This disparity may reflect factors such as genetic isolation in NW begomoviruses and host-specific evolution in OW viruses (Briddon et al., 2010b).

In addition to helper geminiviruses, DNA satellites also provide important insights into viral evolution. Alphasatellites are believed to have originated from the Rep-encoding elements of nanovirus genomes. This highlights out the significance of small replicons in viral evolution (Dolja et al., 2020). Conversely, evolutionary exchange between geminiviruses and other mobile genetic elements (MGEs) may be bidirectional. Evidence suggests that geminiviruses can lose their CP gene and revert to a capsidless, plasmid-like MGE. This reciprocal relationship may help explain the origin of some phytoplasmal plasmids (Kazlauskas et al., 2019; Krupovic et al., 2019).

Ultimately, the contemporary geminivirus evolution is shaped by high mutation rates, rapid inter- and intraspecific recombination and strong selection pressures (Nigam, 2021). These factors facilitate host-range expansion, resistance breaking (as seen in TYLCV) and the emergence of severe disease complexes. These processes are often exacerbated by synergistic DNA satellites such as betasatellites in CLCuD (Briddon, 2003; Mansoor et al., 2003; Prabhandakavi et al., 2020; Prabhandakavi et al., 2021).

Collectively, current evidence supports a modular evolutionary model in which geminiviruses have evolved through recombination-driven assembly of distinct functional components. Replication-associated proteins likely originated from ancestral circular DNA elements, whereas CP may have evolutionary links to RNA viruses or insect-associated viral lineages. The emergence of bipartite genomes, particularly the DNA-B component, may represent a later evolutionary adaptation that improved viral movement and host interactions. This model supports a non-linear evolutionary trajectory in which gene exchange, recombination, and ecological factors collectively drive geminivirus diversification.

### 9.1 Future perspectives

Despite considerable progress in understanding geminivirus biology, several major questions remain unresolved. We propose a “replicon-first” model, in which a circular DNA replicon acquired a functional CP after establishing RCR. The acquisition of CP may have resulted in increased extracellular stability and vector adaptation. Alternatively CP may have been recruited through recombination or horizontal genetic exchange. These models suggest the possible existence of intermediate Rep-containing capsid-less elements and mosaic evolutionary relationships between CP and Rep. Future research should integrate multi-omics approaches with long-read sequencing, population genomics, vector biology, and functional validation to obtain a more comprehensive view of virus–host–vector interactions and evolutionary processes. Structural and functional studies of key viral proteins, particularly Rep, CP, movement-associated proteins, and satellite-encoded pathogenicity factors, will be essential for clarifying their origins, functional convergence, and lineage-specific diversification. In addition, integrating virus–vector interaction studies with climate, cropping-system, and trade-network data will improve predictions of viral emergence and spread. Such efforts will refine current evolutionary models and support more durable disease-management strategies, including resistance breeding, vector management, and surveillance-based early warning systems.

## Footnotes

1 Available at http://gpi.geminiviridae.com/

2
https://www.ncbi.nlm.nih.gov/labs/virus/vssi/#/
