# Supplementary material for: Geminiviruses and their satellites: genomic diversity, evolutionary insights, and emerging models
Source: Front Microbiol. 2026 Oct 12;17:1947355. doi: 10.3389/fmicb.2026.1947355 (PMC13696870; doi:10.3389/fmicb.2026.1947355)
Supplement: Supplementary file 1 [file Data_Sheet_1.DOCX]

**Methodology for regional taxonomic richness and database record analysis**

To calculate the geographically documented taxonomic composition of geminiviruses and their associated satellites, data was retrieved from NCBI Virus (<https://www.ncbi.nlm.nih.gov/labs/virus/vssi/>), and their identities were mapped to the International Committee on Taxonomy of Viruses (ICTV) taxonomy. For this purpose, ICTV Master Species List 41 (MSL41) was used which was released in 2025 by ICTV and ratified in February 2026 (ICTV, 2025). Moreover, Virus Metadata Resource (VMR) current version (VMR_MSL.v1.20260729) was also downloaded from ICTV (ICTV, 2026). In this mapping, isolates and strains were treated as records belonging to the same taxon. Duplicate records were removed and only distinct records were retained for analysis. Thus, number of NCBI records representation is distinct from the taxonomic richness.

Geographic information of records was obtained from NCBI metadata. Countries mentioned in these records were mapped to any of these pre-defined eight centers(Nawaz-ul-Rehman and Fauquet, 2009): A– Australia, B – Japan, C – China, D – Indian subcontinent, E – Africa, F – Mediterranean-European region, G – Central America, H – South America. Moreover, if a record has no geographic information mentioned, it was excluded from analysis dataset as 77 records were excluded on this basis from betasatellites dataset.

Most importantly, when a species was represented in records belonging to different countries considered in one geographic center, that species was counted once within that center for species richness by center. However, the global species richness was calculated from the unique species across the complete dataset. Therefore, in calculations, regional species-richness totals are not expected to sum to global species richness.

Species richness and genus richness were calculated by formulas as: S= number of unique taxonomic species represented in the dataset and genus’s richness=number of unique genera represented in dataset. NCBI database record count was done as number of unique accession records. In this way, repeated isolates/strains of a same species in a region increased database record count but did not increase species richness unless they represented a taxonomically distinct species.

Number of records retrieved from database were 16,872 for geminiviruses, 2279 for alphasatellites and 3256 for betasatellites. Excluded records in the dataset were mentioned separately in each analysis file. 94 records were excluded from the geminiviruses calculation as 93 records have no geographic information and 1 record lacked a standardized species taxonomy. 73 records were excluded from alphasatellites information set as their association with *Nanoviridae* was confirmed. As mentioned earlier, 77 records were excluded from betasatellites dataset as they lacked geographic information.

**References**

Ictv (2025). ICTV Master Species List (MSL41). Available: <https://ictv.global/taxonomy>.

Ictv (2026). Virus Metadata Resource (VMR_MSL41.v1.20260729). Available: <https://ictv.global/vmr>.

Nawaz-Ul-Rehman, M.S., and Fauquet, C.M. (2009). Evolution of geminiviruses and their satellites. FEBS Lett. 583**,** 1825-1832.
