# Supplementary material for: Geminiviruses and their satellites: genomic diversity, evolutionary insights, and emerging models
Source: Front Microbiol. 2026 Oct 12;17:1947355. doi: 10.3389/fmicb.2026.1947355 (PMC13696870; doi:10.3389/fmicb.2026.1947355)
Supplement: Supplementary file 2 [file Table_2.DOCX]

**Supplementary Table S2. Comparative evaluation of proposed evolutionary hypotheses for geminiviruses.**

| **Potential Organism** | **Core Concept/ Mechanism** | **Supporting Evidence / Key Findings** | | **Contradictory evidence** | | **Major limitation /missing link** | | **Current level of geminivirus support** | |
| --- | --- | --- | --- | --- | --- | --- | --- | --- | --- |
| Phytoplasma | Phtoplasmas and RNA viruses co-infected a plant and phytoplasma’s plasmid acquired CP gene from RNA virus, giving rise to ancestors of modern day geminiviruses (Krupovic et al., 2009). Geminivirus may have evolved from rolling-circle plasmids of phytoplasma or algae (Nash et al., 2011) | Geminiviral Coat protein (CP) has similar jelly-roll fold like those of icosahedral ssRNA viruses (Satellite tobacco necrosis virus).  Moreover, phylogenetic and clustering analysis show that geminiviral Rep proteins and phytoplasmal Reps share a common ancestor (Krupovic et al., 2009).  Moreover, geminiviruses and phytoplasmas are phloem limited in infected plants (Krupovic et al., 2009) and both share leafhoppers as one common vector (Christensen et al., 2005;Rojas et al., 2005). Geminivirus Rep sequence (GRS) is found in Rep proteins of geminiviruses, phytoplasmas and of algae | | Phylogenetic, compositional and functional analyses have shown that extrachromosomal DNA (EcDNA) encoded Rep in phytoplasmas is acquired from geminiviruses through a horizontal gene transfer rather than geminiviruses evolving from phytoplasmal EcDNA (Saccardo et al., 2011).  This theory is also challenged by different subcellular compartments of satellite tobacco necrosis virus like RNA viruses and geminiviruses (Saccardo et al., 2011) | | No transitional non-geminate virus containing geminiviral Rep paired with non- geminivirus capsid has been found on land plants (Saccardo et al., 2011). | | Low | |
| Unknown virus | Squash leaf curl virus lineage (S-Lin) begomoviruses may have been evolved through recombination between a NW begomovirus and a virus from an unknown lineage (Torres-Herrera et al., 2019) | Similarities between ~670bp genome module containing AC4 gene and 480bp of Rep gene support this theory (Torres-Herrera et al., 2019) | -------------- | | No homolog of S-Lin Rep or AC4 gene has been found in any public database or detected in any examined endogenous viral elements “fossils” | | Moderate | |  |
| Algae or phytoplasma | Geminivirus may have evolved from rolling-circle plasmids of phytoplasma or algae (Nash et al., 2011). Another study proposed that ssDNA in red algae acquired new genes over time (~450M years ago) and transitioned towards a “pre-mastrevirus” lineage (Nawaz-ul-Rehman and Fauquet, 2009) | Geminivirus Rep sequence (GRS) is found in Rep proteins of geminiviruses, phytoplasmas and of algae (Nash et al., 2011).  Sequence homology (>31% over 55 amino acids)between Rep protein of Wheat dwarf virus and plasmid of *Porphyra pulchera* provided additional evidence for relationship between algae and geminiviruses (Nawaz-ul-Rehman and Fauquet, 2009) | GRS motif is not found in Rep proteins of nanoviruses and circoviruses (Krupovic et al., 2009;Nash et al., 2011) | | The central loop of GRS remain uncharacterized(Nash et al., 2011) | | Moderate | |  |
| Aniosperms | Rep genes of geminiviruses and of angiosperms are coevolving (Sharma et al., 2020).  Phloem limitation of some geminiviruses reflects coevolution with angiosperm phloem (Rojas et al., 2005). | In 42 angiosperm species, endogenous geminivirus like elements (EGV) are homologues of geminiviruses Rep genes (Sharma et al., 2020).  Replication, movement and transmission of some monopartite begomoviruses and curtoviruses is limited to phloem (Rojas et al., 2005). |  | |  | |  | |  |
| Insect vector | CP of geminiviruses are co-evolving with their insect vectors (Lefeuvre et al., 2019;Dolja et al., 2020) | Phylogeny of 276 sequence dataset of geminiviruses was broadly congruent with the vector genera (Lefeuvre et al., 2019) | *Mastrevirus* is an exception. Its CPs are so diverse that they fall into seven different clusters (Lefeuvre et al., 2019) | | Phylogeny was built on a limited set of nine vector genera. Thus, broader vector sampling and denser viral taxon sampling are needed to determine the generality of virus–vector coevolution. | | Strong | |  |
| RNA viruses | Geminiviral CP may have evolved from CP of an icosahedral ssRNA virus (satellite tobacco necrosis virus or its related ancestor) (Krupovic et al., 2009;Dolja et al., 2020) | CPs of geminiviruses share structural similarity with the CP of satellite tobacco necrosis virus (Krupovic et al., 2009) | Structural similarity alone does not establish the direction or mechanism of evolutionary transfer. The proposed RNA-virus origin is also challenged by the intracellular compartmentalization of RNA-virus and geminivirus replication (Saccardo et al., 2011). | | No intermediate found | | Moderate | |  |

**References**

Christensen, N.M., Axelsen, K.B., Nicolaisen, M., and Schulz, A. (2005). Phytoplasmas and their interactions with hosts. Trends Plant Sci. 10, 526-535.

Dolja, V.V., Krupovic, M., and Koonin, E.V. (2020). Deep roots and splendid boughs of the global plant virome. Annu. Rev. Phytopathol. 58, 23-53.

Krupovic, M., Ravantti, J.J., and Bamford, D.H. (2009). Geminiviruses: a tale of a plasmid becoming a virus. BMC Evol. Biol. 9:112. doi: 10.1186/1471-2148-9-112.

Lefeuvre, P., Martin, D.P., Elena, S.F., Shepherd, D.N., Roumagnac, P., and Varsani, A. (2019). Evolution and ecology of plant viruses. Nat. Rev. Microbiol. 17, 632-644.

Nash, T.E., Dallas, M.B., Reyes, M.I., Buhrman, G.K., Ascencio-Ibañez, J.T., and Hanley-Bowdoin, L. (2011). Functional analysis of a novel motif conserved across geminivirus Rep proteins. J. Virol. 85, 1182-1192.

Nawaz-Ul-Rehman, M.S., and Fauquet, C.M. (2009). Evolution of geminiviruses and their satellites. FEBS Lett. 583, 1825-1832.

Rojas, M.R., Hagen, C., Lucas, W.J., and Gilbertson, R.L. (2005). Exploiting chinks in the plant's armor: evolution and emergence of geminiviruses. Annu. Rev. Phytopathol. 43, 361-394.

Saccardo, F., Cettul, E., Palmano, S., Noris, E., and Firrao, G. (2011). On the alleged origin of geminiviruses from extrachromosomal DNAs of phytoplasmas. BMC Evol. Biol. 11:185. doi: 10.1186/1471-2148-11-185.

Sharma, V., Lefeuvre, P., Roumagnac, P., Filloux, D., Teycheney, P.Y., Martin, D.P., and Maumus, F. (2020). Large-scale survey reveals pervasiveness and potential function of endogenous geminiviral sequences in plants. Front. Microbiol. 6:veaa071. doi: 10.1093/ve/veaa071.

Torres-Herrera, S.I., Romero-Osorio, A., Moreno-Valenzuela, O., Pastor-Palacios, G., Cardenas-Conejo, Y., Ramírez-Prado, J.H., Riego-Ruiz, L., Minero-García, Y., and Ambriz-Granados, S. (2019). A lineage of begomoviruses encode rep and AC4 proteins of enigmatic ancestry: hints on the evolution of geminiviruses in the New World. Viruses 11:644. doi: 10.3390/v11070644.
